# Conquering the Synthesis and Functionalization of Bicyclo[1.1.1]pentanes

**DOI:** 10.1021/jacsau.3c00014

**Published:** 2023-05-16

**Authors:** Bethany R. Shire, Edward A. Anderson

**Affiliations:** Chemistry Research Laboratory, Department of Chemistry, 12 Mansfield Road, Oxford OX1 3TA, United Kingdom

**Keywords:** Bicyclo[1.1.1]pentane, Bioisostere, [1.1.1]Propellane, Radical reactions, Photoredox catalysis

## Abstract

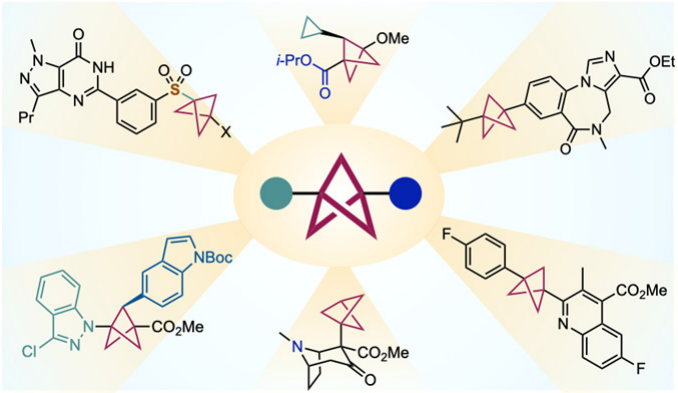

Bicyclo[1.1.1]pentanes (BCPs) have become established as attractive bioisosteres for *para*-substituted benzene rings in drug design. Conferring various beneficial properties compared with their aromatic “parents,” BCPs featuring a wide array of bridgehead substituents can now be accessed by an equivalent variety of methods. In this perspective, we discuss the evolution of this field and focus on the most enabling and general methods for BCPs synthesis, considering both scope and limitation. Recent breakthroughs on the synthesis of bridge-substituted BCPs are described, as well as methodologies for postsynthesis functionalization. We further explore new challenges and directions for the field, such as the emergence of other rigid small ring hydrocarbons and heterocycles possessing unique substituent exit vectors.

## Introduction

**Figure 1:**
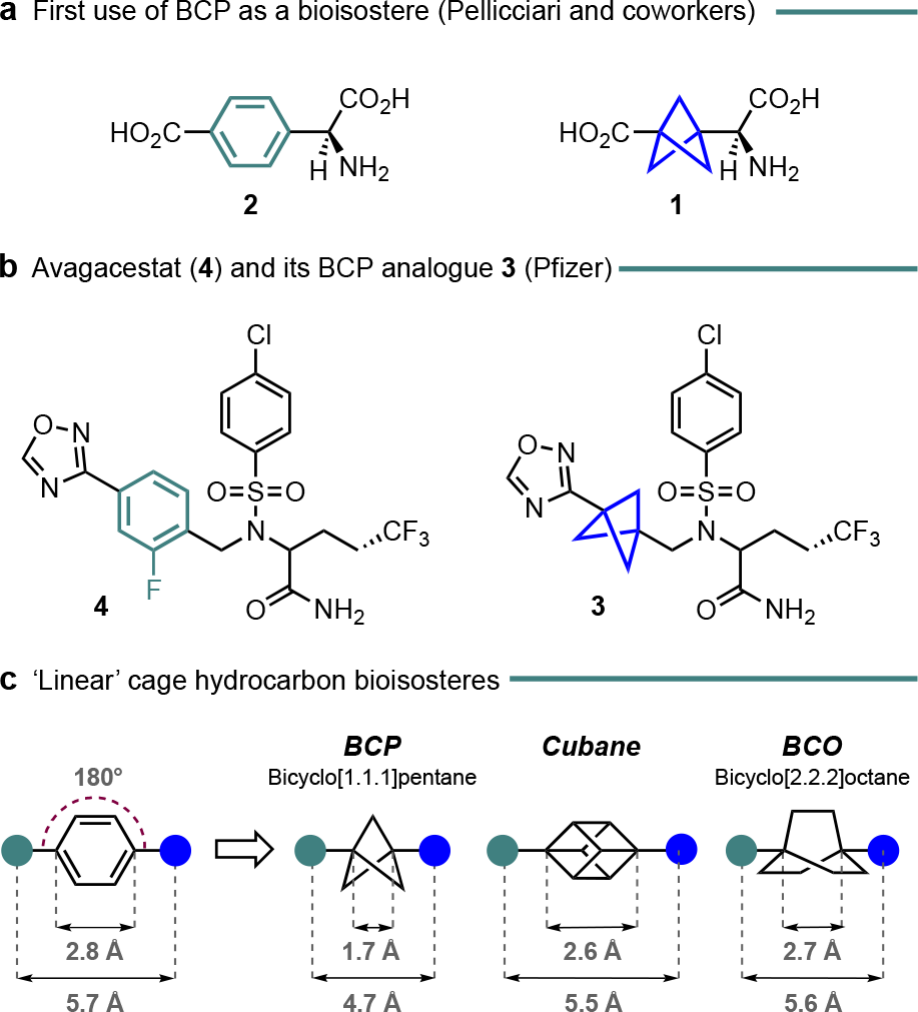
(a,b) Seminal examples of bicyclo[1.1.1]pentanes (BCPs) as *para*-substituted arene bioisosteres.^3,4^ (c) Dimensions of the BCP and other cage hydrocarbons as linear bioisosteres.

The quest for molecular scaffolds that improve the physicochemical and pharmacokinetic properties of drug candidates has inspired the invention of numerous functional group bioisosteres over many decades.^1^ Finding suitable surrogates for benzene rings has presented a particular challenge in this field,^2^ as few chemical motifs are able to mimic the geometry and substituent exit vectors of a benzene ring, while also offering the desired property benefits. A possible solution to this challenge emerged in 1996, when Pellicciari and co-workers described the synthesis of a bicyclo[1.1.1]pentane (BCP) analogue **1** of (*S*)-(4-carboxyphenyl)glycine **2** (Figure 1a),^3^ which showed selective and potent activity as an antagonist of the metabolic glutamate receptor mGluR1. Although the group noted the structural overlay of the two compounds, this finding was largely overlooked outside of this field for over a decade, and the potential of the BCP remained unrealized. It was not until 2012 that interest in this field was truly kindled with the report by Stepan and colleagues at Pfizer of a BCP analogue **3** of the γ-secretase inhibitor avagacestat **4** (Figure 1b).^4^ This compound not only exhibited equivalent biological activity to the parent drug but, importantly, also displayed enhanced solubility, membrane permeability, and reduced metabolic susceptibility. In a period where “escape from flatland” was fast becoming a key concept in drug design,^5^ the introduction of this sp^3^-rich scaffold as a potential bioisostere for *para*-substituted benzene rings was a highly attractive prospect not only chemically but also from an intellectual property perspective.

Since that report, there has been an explosion of interest in this remarkable rigid scaffold, with over 250 papers involving BCPs in the primary chemical literature, and over 10 000 BCPs described.^6^ Where biological properties have been reported, a number of the BCPs have also been demonstrated to improve the pharmacological profiles of drug candidates, although in some cases this is not accompanied by maintained bioactivity; for such examples, it is likely the benzene ring serves as more than a simple “spacer” unit and instead engages in π–π interactions or other binding modes that cannot apply to the three-dimensional structure of BCPs.

Structurally, bicyclo[1.1.1]pentanes have primarily been deployed as substitutes for commonplace *para*-substituted arenes. The bridgehead substituents perfectly replicate the 180° exit vector of the *para*-arene, albeit with ∼1 Å shorter substituent separation (Figure 1c). While hydrocarbons such as cubane and bicyclo[2.2.2]octane more accurately mimic the substituent separation, they are significantly harder to access with diversity at the bridgehead positions and, in the latter case, do not bring the same benefits to physicochemical properties. BCPs are less-commonly utilized as bioisosteres for alkynes (now exhibiting lengthened substituent separation) and also *tert*-butyl groups. Although BCPs are also commonly referred to as “strained” and possess around 66.6 kcal mol^–1^ of ring strain energy,^7^ they are nonetheless generally kinetically inert toward ring-opening reactions and, as noted above, are resistant toward metabolic degradation.

In this perspective, we discuss some of the most important developments in the synthesis of BCPs over the past decade and cover novel and general methods for the synthesis and functionalization of this important framework, including work from our own group. We also consider new directions and challenges for this evolving field as cage hydrocarbons and related heterocycles move from their role as arene mimics to useful and distinct molecular cores in their own right. As with any such article, we do not aim to provide a comprehensive coverage of the extensive BCP literature, for which the reader is referred to a selection of complementary reviews.^2b,8^

## Conceptual Approaches to Bicyclo[1.1.1]pentanes

**Scheme 1:**
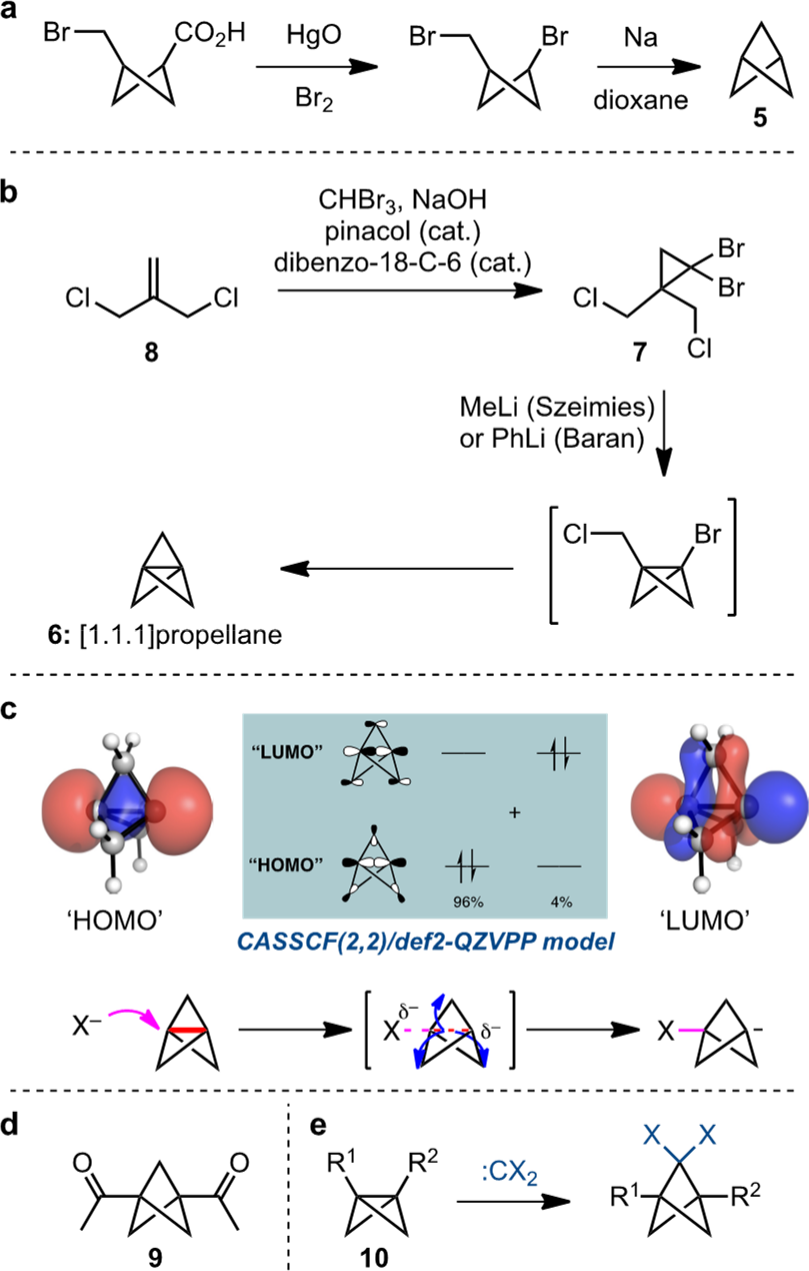
Wiberg Synthesis of Bicyclo[1.1.1]pentane,^9^ (b) the Optimal Synthesis of [1.1.1]Propellane,^11,13^ (c) a Delocalized Ground-State Description of [1.1.1]Propellane Connects to Its Reactivity with Anions and Radicals, (d) Biacetyl BCP, and (e) Carbene Addition to Bicyclo[1.1.0]butanes

Bicyclo[1.1.1]pentane itself (**5**, Scheme 1a), was first synthesized in 1964 by Wiberg and co-workers.^9^ At that time, the molecule represented something of a curiosity and certainly a significant synthetic challenge. Comprising a cyclobutyl-bridged cyclobutane, BCP possesses 66.6 kcal mol^–1^ of strain energy compared with the straight-chain hydrocarbon pentane but was nonetheless found to be a robust functional group. Over the next 40 years, a number of other routes emerged for the preparation of BCPs, but it was not until the synthesis of the strained hydrocarbon [1.1.1]propellane (**6**, Scheme 1b) was described in 1982 by Wiberg and Walker that a major breakthrough was achieved.^10^ [1.1.1]Propellane is, itself, a fascinating molecule from a structural perspective, but it is its ability to react with a variety of reagents to directly afford BCPs that has arguably revolutionized its chemistry. The most popular route to [1.1.1]propellane, developed by Szeimies and co-workers,^11^ begins with the synthesis of dibromocyclopropane **7** by cyclopropanation of dichlorobutene **8** (modified conditions for this process developed by Lynch and Dailey are shown).^12^ Sequential bromine–lithium exchange from **7**, with each carbanion undergoing cyclization onto its proximal chloromethyl substituent, affords [1.1.1]propellane.^11^ The finding by the Baran group that phenyllithium is sufficiently reactive to effect this process has improved the yield, scalability, and purity of the resulting propellane,^13^ which can be stored as solution (typically in ethereal solvents) for several months without significant degradation. Because, in part, of its unique bonding, [1.1.1]propellane is able to undergo ring-opening reactions with anions, radicals, and cations, with the former two delivering BCP products. Often termed “strain-release” additions, our group has proposed that these processes in fact benefit from transition state stabilization by the delocalization of electron density from the breaking C–C bond onto the bridging carbon atoms. Specifically, one model for the ground state of [1.1.1]propellane describes its central bond as partly occupying the next highest energy molecular orbital (formerly the LUMO in a localized bonding model), which has the benefit of reducing Pauli repulsion within the propellane cage. The addition of further electron density to the propellane, as occurs during anionic ring-opening, can be accommodated by cage compression in order to increase the extent of electronic delocalization at the transition state.

As will be seen, [1.1.1]propellane certainly offers a versatile entry to many BCPs, but it is not without its drawbacks. Chief among these are the difficulty of its storage and transport, especially on large scales, and also the need for two equivalents of alkyl- or aryllithium reagents for its synthesis. As a result, the availability of preformed, difunctionalized BCPs, such as “biacetyl BCP” **9**, have also served as a popular entry point to BCP derivatives. However, these starting materials frequently require lengthy synthetic sequences to attain target compounds and, as such, will not be discussed extensively here. Nonetheless, from an industrial perspective, this strategy is certainly appealing in avoiding the need to synthesize and handle **6**.

**Scheme 2:**
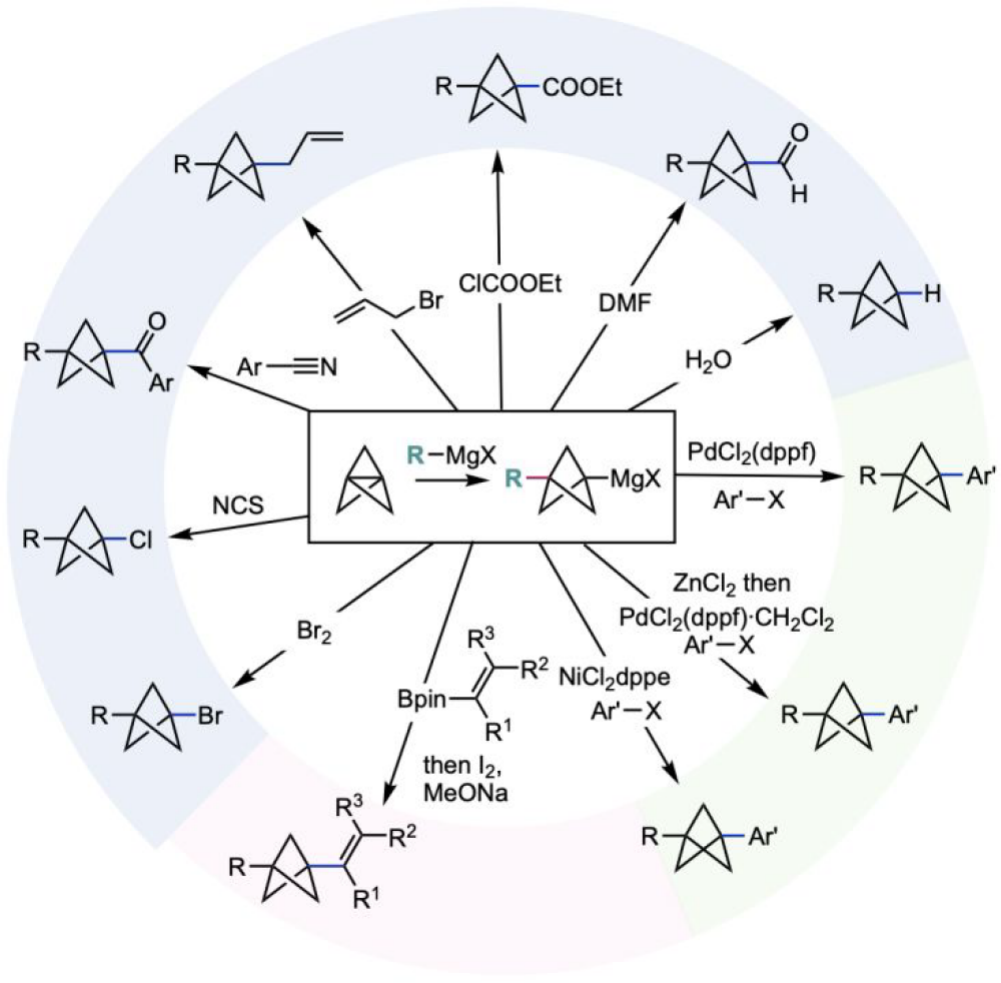
Formation and Reactions of BCP Grignard Reagents (de Meijere et al.^16a^ and Knochel et al.^16b^)

Fortunately, other methods for BCP synthesis offer potential solutions to this challenge. Recent developments will be discussed later in this perspective; here we therefore, only note the most popular of these tactics, namely the insertion of (dihalo) carbenes into the interbridgehead bond of bicyclo[1.1.0]butanes (BCBs, **10**, Scheme 2e). First described by Applequist and co-workers,^14^ this chemistry has been deployed in industry settings,^15^ but does suffer from the drawback of requiring radical-based dehalogenation (typically achieved with tin hydride or silane reagents) if the parent methylene-bridged BCP is required.

## BCPs from Anionic Addition to [1.1.1]Propellane

**Scheme 3:**
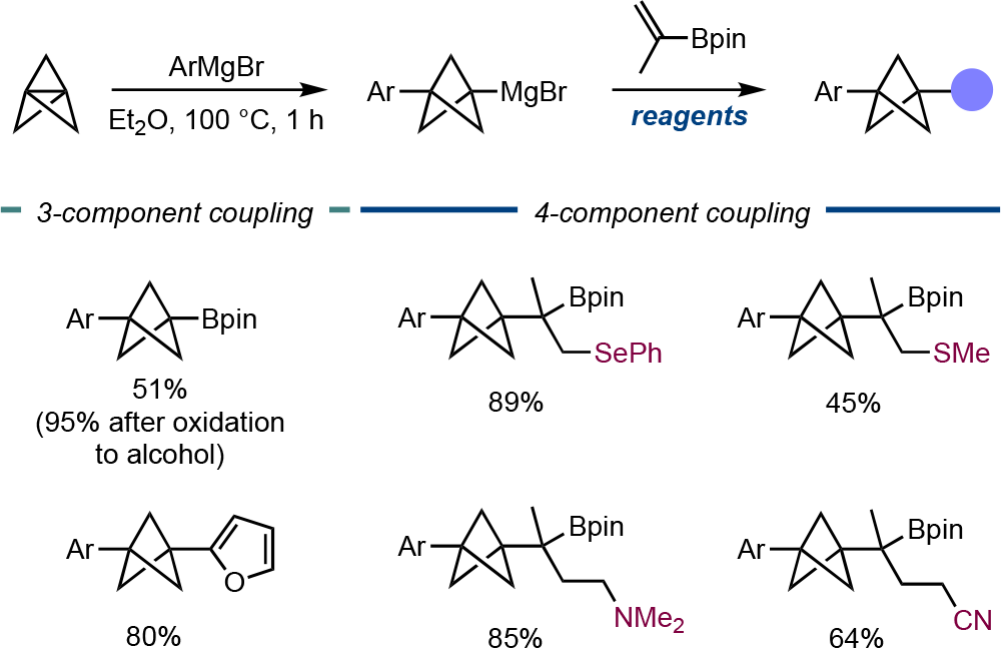
*In Situ* Grignard Formation/Borylation/1,2-Metallate Rearrangement to Form Ar/C-Disubstituted BCPs (Aggarwal et al.^17^)

A popular entry to BCPs from [1.1.1]propellane is the addition of carbon- and heteroatom-centered anions, followed by either protic quenching to form terminal BCPs or further reaction to form disubstituted BCPs. The use of highly reactive organometallics and relatively harsh reaction conditions are generally a feature of these reactions (e.g., aryl Grignard addition requires heating to 100 °C in diethyl ether, which requires sealed tube conditions and presents associated safety implications). This can, in turn, limit functional group tolerance; however, the resulting BCP organometallics have been shown to be versatile building blocks for further functionalization through electrophilic trapping or cross-coupling. In spite of these limitations, the use of alkyl and aryl Grignard reagents to form carbon-substituted BCPs has become established as a highly useful approach to BCPs, with a wide range of further functionalizations having been demonstrated (Scheme 2).^16^ Halide, carbonyl, and allyl groups have all been installed through reaction with appropriate electrophiles, as well as both palladium- and nickel-catalyzed cross-coupling with aryl and heteroaryl halides.^16^ Recently the Aggarwal group expanded this addition chemistry to synthesize highly substituted BCPs via 1,2-metalate rearrangement of BCP-boronate complexes formed upon reaction of the BCP Grignard adducts with vinyl boronic esters (Scheme 3).^17^ A variety of electrophiles and radicals were engaged with the intermediate complex, which resulted in three- and four-component coupling processes to form a wide variety products still bearing the useful Bpin group. This chemistry represents a powerful, modular method for the synthesis of diverse and highly substituted BCPs with multiple points of diversification.

**Scheme 4:**
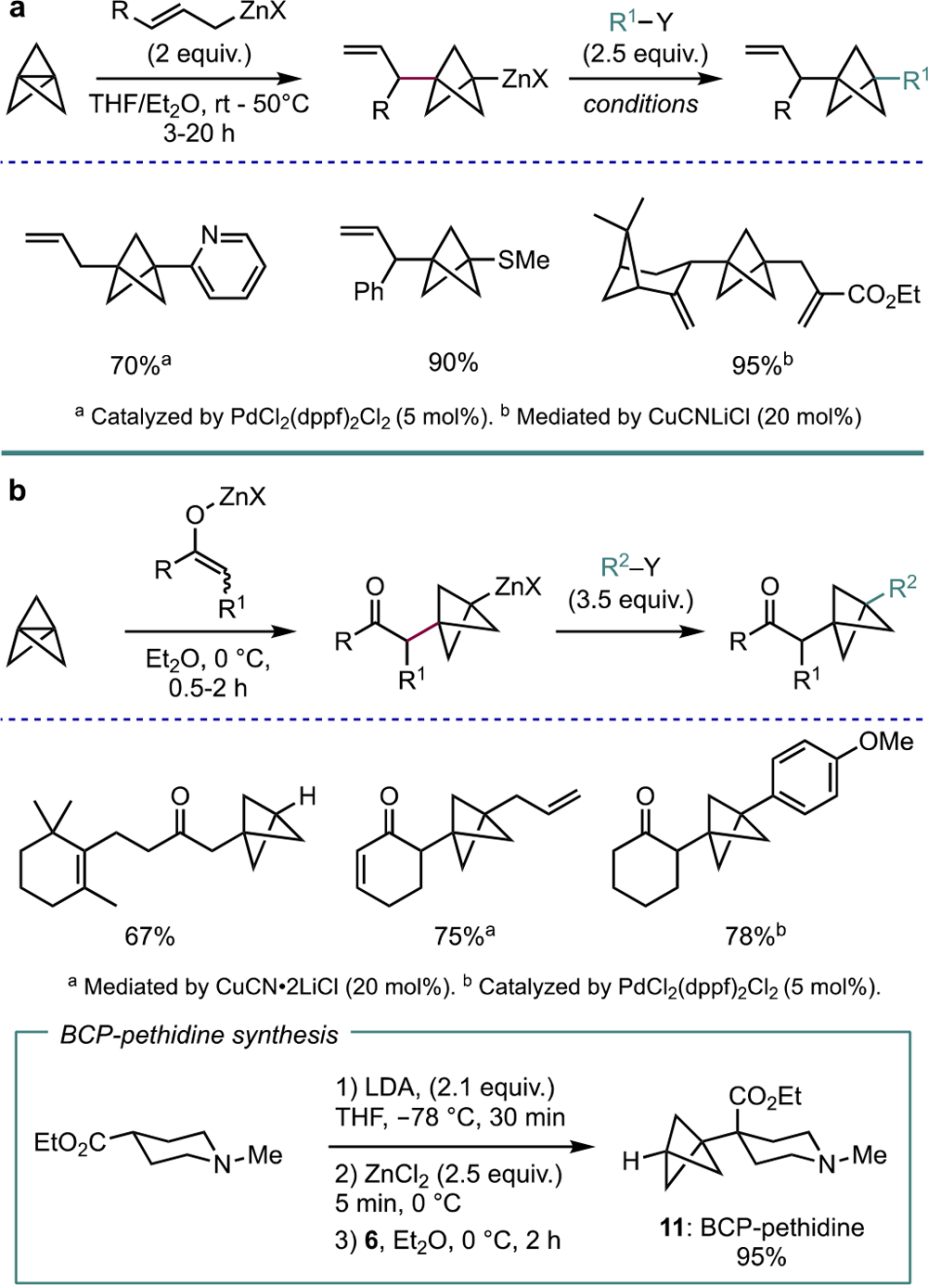
Synthesis of Disubstituted BCPs from the Addition of (a) Allylzinc or (b) Zinc Enolate Species to [1.1.1]Propellane (Knochel et al.^18^)

The Knochel group, who had previously contributed an efficient Negishi cross-coupling of aryl-BCP Grignard adducts,^16b^ recently discovered a highly regioselective addition of allylzincs and zinc enolates to [1.1.1]propellane that, similarly to BCP Grignards, could then be submitted to electrophilic trapping or Negishi cross-coupling (Scheme 4).^18^ It is notable that the enolate addition protocol (Scheme 4b) proceeds at 0 °C, which is in contrast to the typical need for heating in anionic addition processes. As well as forming a wide range of aliphatic, aromatic, and heteroatom-substituted allyl- or α-carbonyl-substituted BCPs, this chemistry also provided access to BCP-pethidine **11**, a synthetic opioid: formation of the zinc enolate species of commercially available ethyl 1-methylpiperidine-4-carboxylate, followed by addition to [1.1.1]propellane, afforded the BCP drug analogue in an impressive 95% yield.

**Scheme 5:**
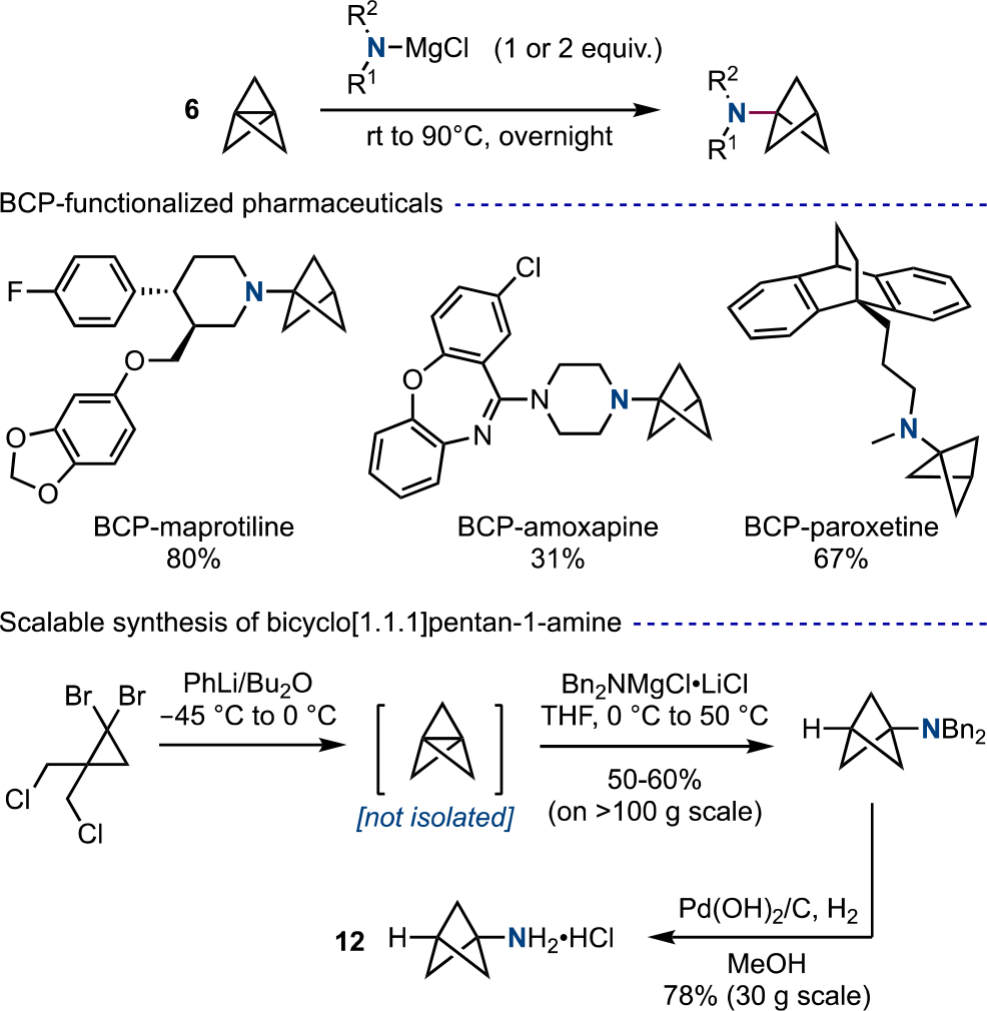
Turbo-Amide Addition to **6** for the Synthesis of Aniline Bioisosteres (Baran et al.^19^)

Nitrogen-centered anions can also be employed in the synthesis of *N*-substituted BCPs. In 2016, Baran and co-workers demonstrated the functionalization of small, strained ring systems, including the central bond of [1.1.1]propellane, using “turbo” amides to form *N*-substituted terminal BCPs under heating (Scheme 5).^13b,19^ In collaboration with Pfizer, this chemistry enabled a scalable and practical synthesis of the valuable BCP-amine building block **12**. More generally, this chemistry offers a convenient one-pot method for the late-stage installation of an aniline bioisostere into drug candidates, rather than requiring multistep approaches from amine **12**, itself, thus rendering the exploration of *N*-substituted BCPs in drug discovery far easier. A subsequent publication from the Gleason group combined turbo amide addition to **6** with copper-catalyzed functionalization of the resulting anion, thus affording *N*,*C*-difunctionalized BCPs.^20^

**Scheme 6:**
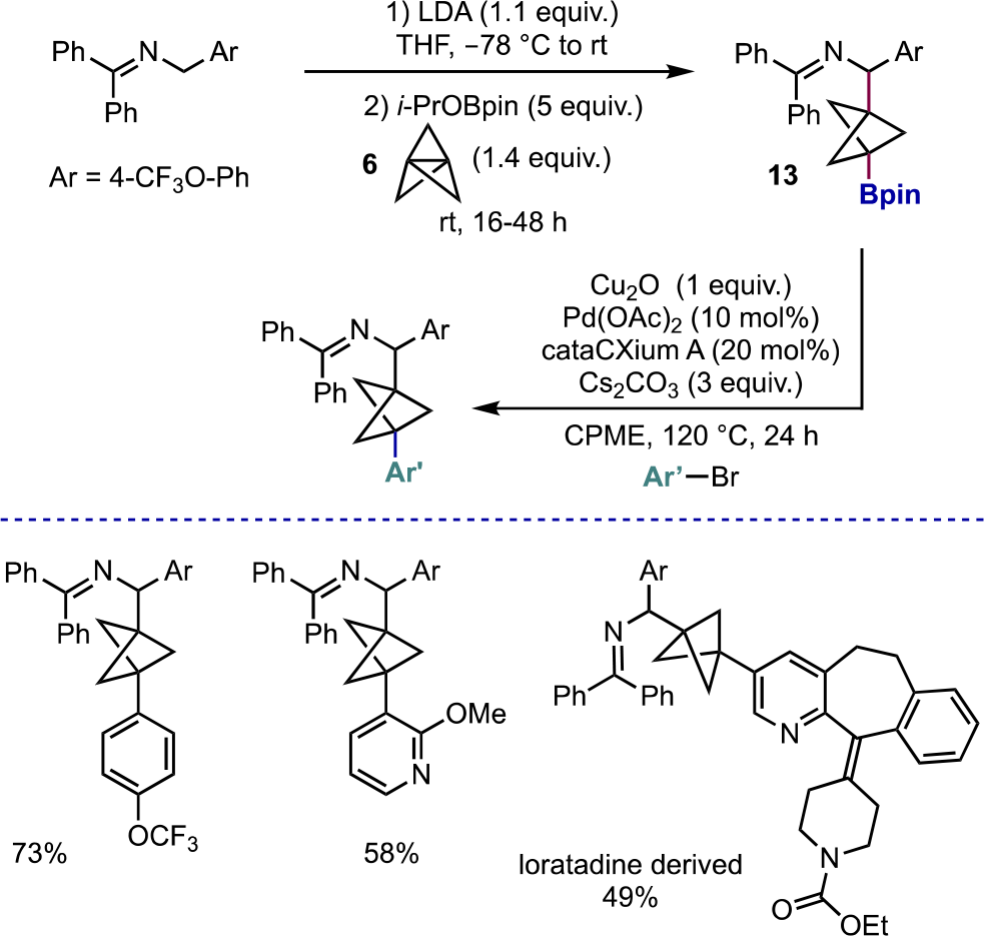
Synthesis and Cross-Coupling of Benzylamine BCP Boronic Esters (Walsh et al.^21^)

Another anionic ring-opening of [1.1.1]propellane that does not require heating to effect reaction was disclosed by the Walsh group, who reported the addition of 2-azaallyl anions to **6** at room temperature to form terminal benzylamine-substituted BCPs (Scheme 6).^21^ They later expanded on this chemistry by trapping the intermediate BCP carbanion with *i*-PrOBpin, thereby affording versatile BCP boronic esters **13**.^22^ This single-step synthesis offers many opportunities for further functionalization, which is something the Walsh group took advantage of in developing a reaction that had previously been absent in BCP functionalization chemistry—a Suzuki cross-coupling that did not require preactivation of the boronate ester or use of additional organometallic reagents. They found that a combination of Pd(OAc)_2_ and cataCXium A as catalyst, alongside stoichiometric Cu_2_O and excess Cs_2_CO_3_, facilitated the cross-coupling of BCP boronic esters with various (hetero)aryl bromides. A notable example is the functionalization of a loratadine-derived aryl bromide, which gave the corresponding BCP in a respectable 49% yield.

## BCPs from Radical Addition to [1.1.1]Propellane

Reactions of [1.1.1]propellane with radical reagents are significantly more common than their anionic counterparts because radicals generally react more readily with [1.1.1]propellane. This can be explained by the abovementioned difference in electron density that must be accommodated inside the propellane cage during radical ring-opening compared with the (less favorable) anionic addition.^23^ As a result, a multitude of strategies have been developed to generate various carbon and heteroatom-centered radicals which, once formed, react efficiently with propellane to form mono- or disubstituted BCPs. The milder reaction conditions and high functional group tolerance render these approaches very useful for late-stage functionalizations and the synthesis of relatively complex druglike BCP-containing compounds in few steps.

**Scheme 7:**
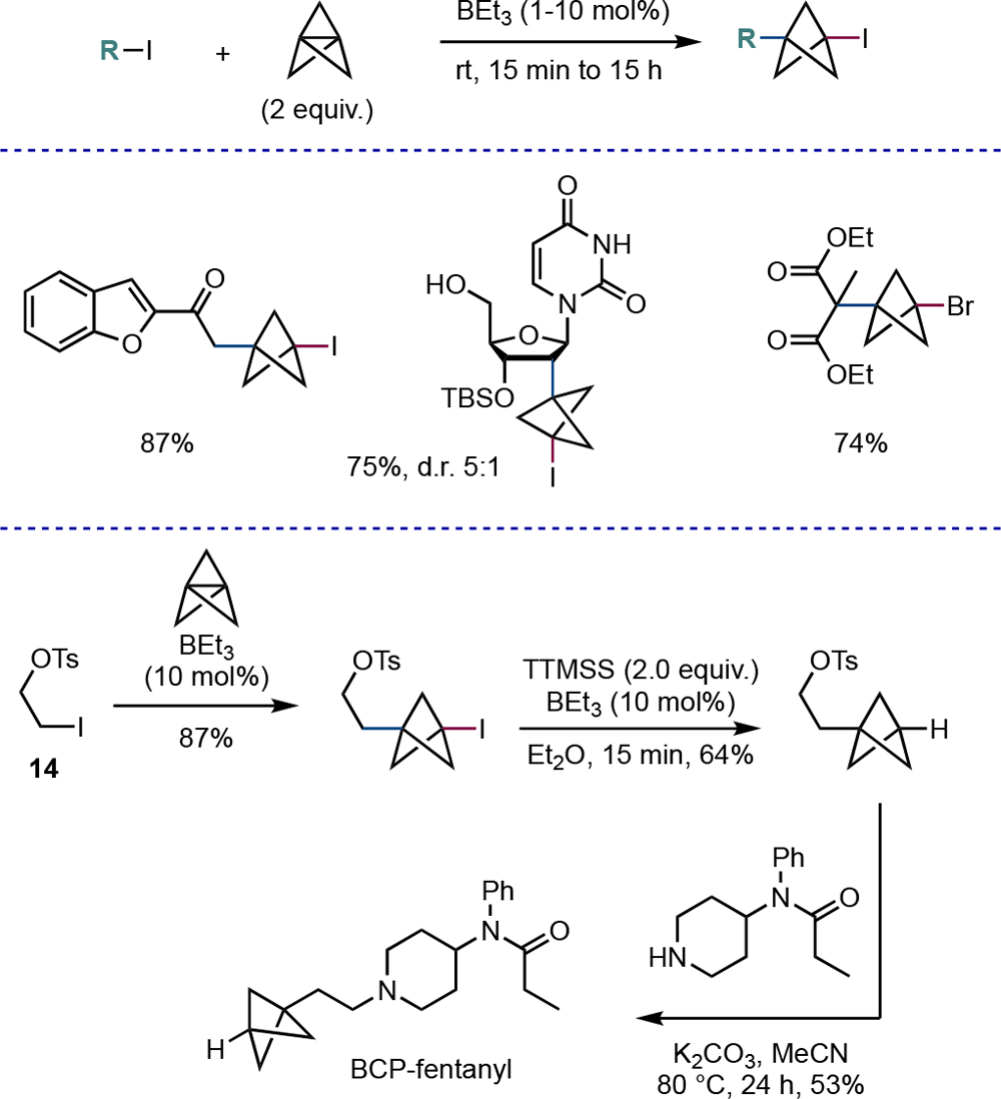
Atom Transfer Radical Addition (ATRA) of Alkyl Halides Using Triethylborane as Initiator (Anderson et al.^25^)

This is also the area of BCP chemistry that our group has largely focused on. We began our foray into the synthesis of BCPs with the aim of synthesizing BCP halides via atom transfer radical addition (ATRA) reactions in order to access products that contained a useful halide handle for further functionalization and diversification. The use of triethylborane as a radical initiator was an appealing alternative to previous methods that had relied on mercury lamp irradiation,^24^ or methyllithium-promoted alkyl halide additions,^16a^ both of which had shown significant functional group limitations and were unsuitable for reactions on scale. We found that a very simple reaction mixture of alkyl halide, [1.1.1]propellane, and (up to) 10 mol % triethylborane proved successful for a wide range of alkyl iodides and bromides with excellent functional group compatibility (Scheme 7).^25^ Use of this method also enabled the synthesis of a BCP analogue of fentanyl in three steps from iodotosylate **14**, although we did note that free amines were not tolerated, potentially because of complexation with the borane initiator.

**Scheme 8:**
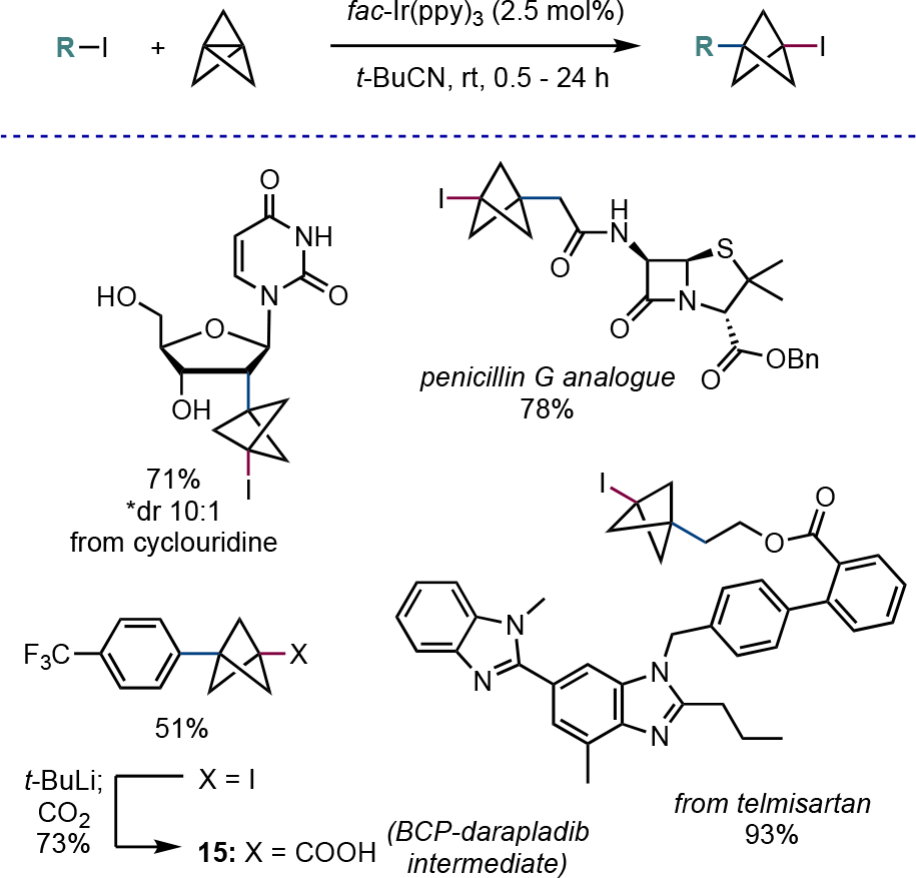
ATRA Addition of Alkyl and (Hetero)aryl Halides under Photoredox Catalysis (Anderson et al.^26^)

Another class of substrates that proved unreactive under triethylborane-initiated ATRA initiation were aryl halides, which prompted us to investigate the use of photoredox methods for radical generation. This proved to be a very successful approach for the formation of BCPs; the use of Ir(ppy)_3_ as catalyst effected ATRA reaction of alkyl, aryl, and heteroaryl halides in high yields with excellent functional group tolerance (Scheme 8).^26^ This method generally proceeded in superior yields to the triethylborane-initiated chemistry and was well-suited for late-stage functionalization of drug derivatives, such as penicillin G and telmisartan. This chemistry also enabled a formal synthesis of BCP-darapladib (first synthesized by GSK),^15^ which forms intermediate **15** in two steps rather than the seven previously required.

**Scheme 9:**
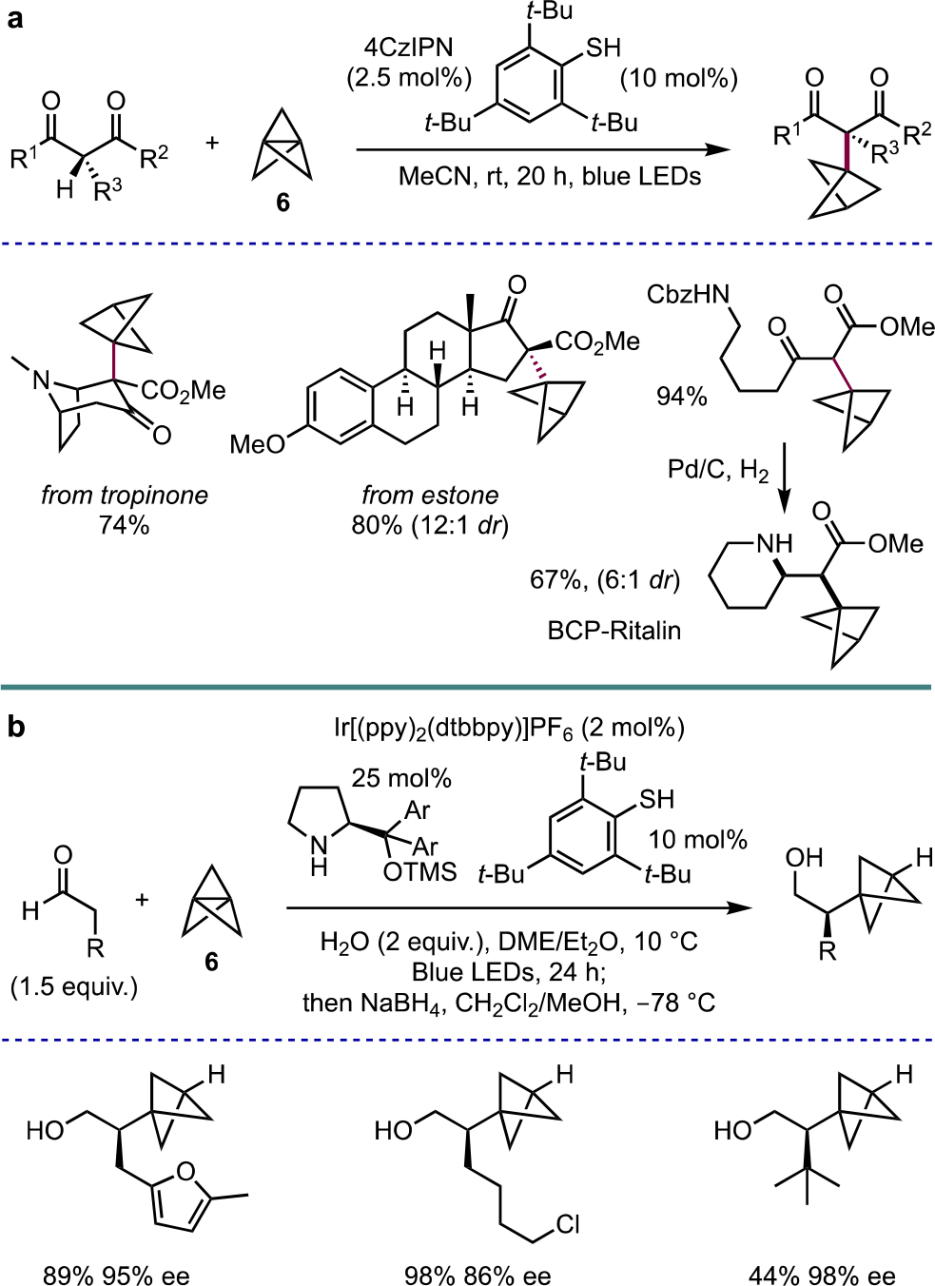
(a) α-Quaternary BCPs Synthesis via Addition of Electron-Deficient Radicals to **2** and (b) α-Chiral BCPs Synthesis via Dual Organo/Photoredox Catalysis (Anderson et al.^27,28^)

Following the success of this methodology, our group developed a number of other photoredox-catalyzed transformations of [1.1.1]propellane to make highly functionalized carbon- and heteroatom-substituted BCPs. Use of the organic photocatalyst 4CzIPN, along with a bulky thiol hydrogen atom source, enabled the synthesis of α-quaternary BCPs through tandem photoredox and hydrogen atom transfer (HAT) catalysis (Scheme 9a).^27^ Functionalization of tropinone and estrone was achieved using this method, as well as the synthesis of the BCP analogue of the ADHD drug ritalin. We were further able to exploit the combination of photoredox catalysis and HAT catalysis, this time together with the Hayashi–Jørgensen diarylprolinol organocatalyst, to perform a direct asymmetric synthesis of α-chiral BCPs from aldehydes and [1.1.1]propellane in excellent yields and ee (Scheme 9b);^28^ this reaction represented the first stereoselective ring opening of **6**.

**Scheme 10:**
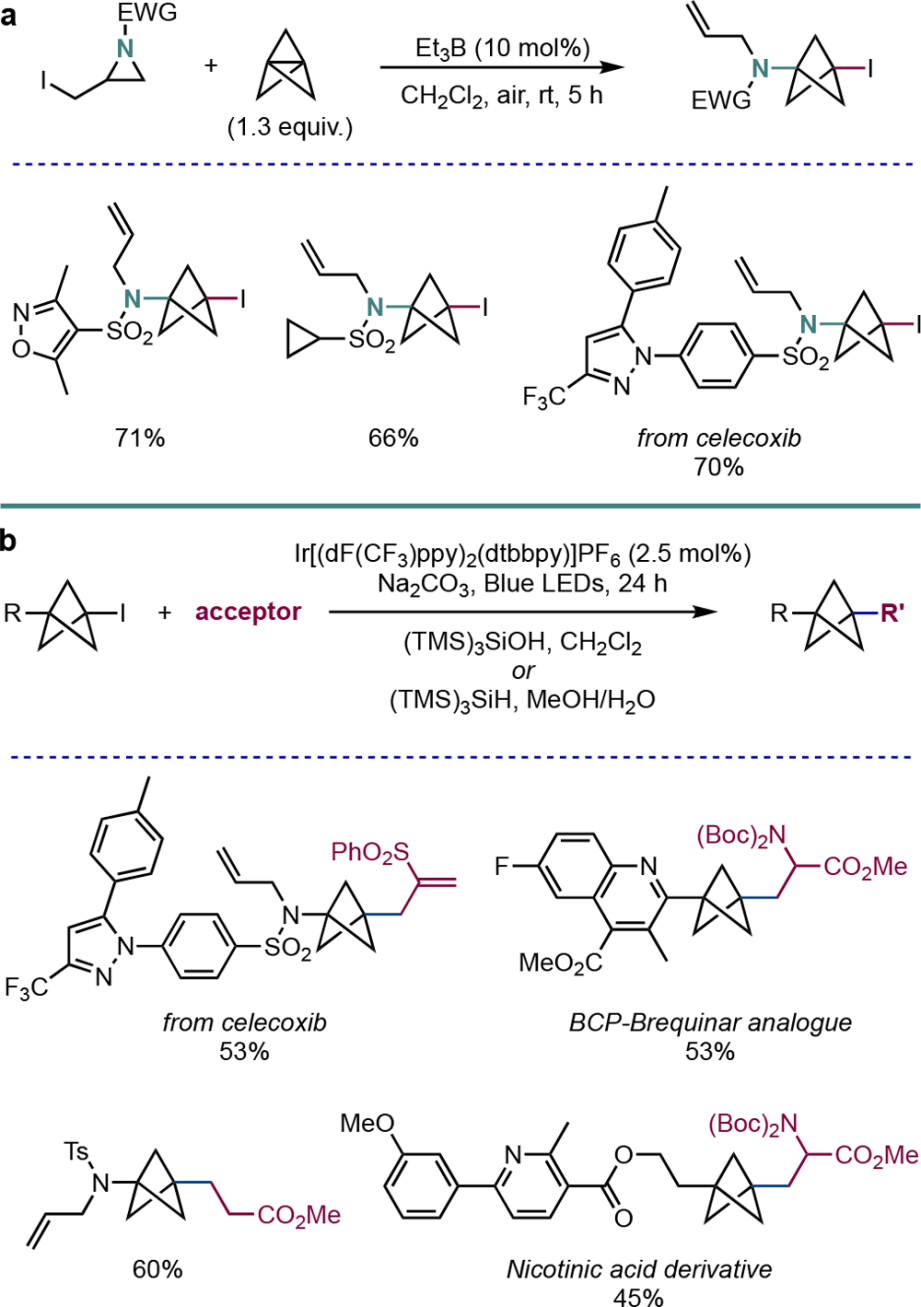
(a) Addition of *N*-Centered Radicals to **2** and (b) Giese Radical-Based Functionalization of BCP Iodides (Anderson et al.^30^)

Our group has also explored the ring-opening of [1.1.1]propellane using heteroatom-centered radicals to form nitrogen- and sulfur-substituted BCPs, as well as their further functionalization. Inspired by seminal work by Taguchi and co-workers on α-iodomethylaziridines,^29^ we found that triethylborane could initiate the formation of *N,I*-substituted BCPs via aziridine fragmentation (Scheme 10a).^30^ Once again, this chemistry could be employed in the functionalization of drug molecules, such as the nonsteroidal anti-inflammatory agent celecoxib.

The products of these reactions, as well as other *C*-substituted BCP iodides, could be further functionalized using Ir{[dF(CF_3_)ppy]_2_(dtbbpy)}PF_6_ as the catalyst in a photoredox-catalyzed Giese reaction (Scheme 10b). The choice of the silane used in this chemistry was critically dependent on the nature of the acceptor, with (Me_3_Si)_3_SiH suitable for acrylates and other species that proceed via capto-stabilized radicals,^31^ but (Me_3_Si)_3_SiOH being appropriate for allyl sulfones and other substrates with “reducible” leaving groups.^32^ Under these two conditions, a wide variety of alkenyl and heteroaryl acceptors proved successful substrates, and the chemistry could be performed on relatively complex BCP examples including derivatives of celecoxib, a nicotinic acid derivative, and a BCP analogue of the dihydroorotate dehydrogenase inhibitor brequinar.

**Scheme 11:**
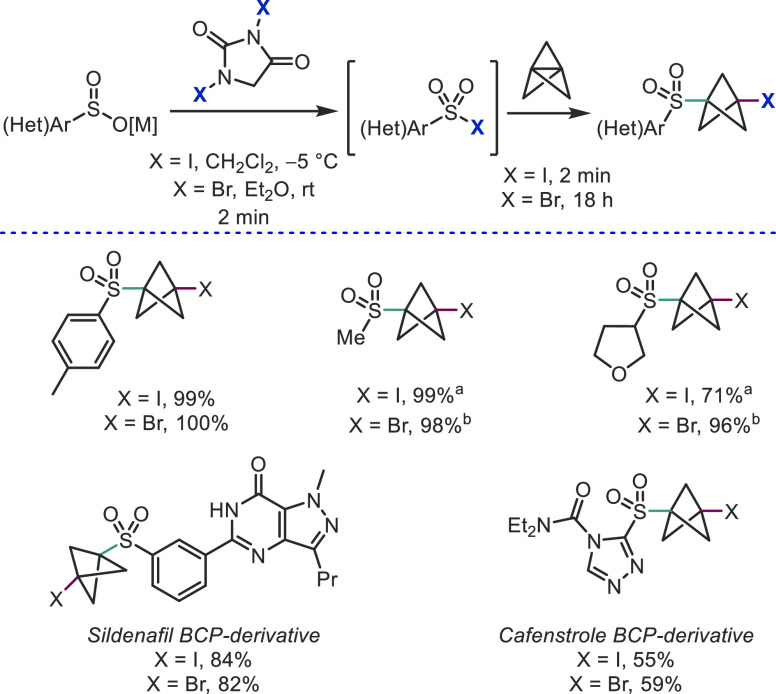
Scalable Synthesis of BCP Sulfones from Sulfonyl Halides (Anderson et al.^33^) BnNMe_3_ICI_2_ was used as iodinating agent. Br_2_ was used as brominating agent, 10 mol % Et_3_B was also added, and the reaction with **6** was performed over 2 h.

Some heteroatom–halide bonds are reactive enough that no radical initiator or photoredox catalyst is necessary at all. On the basis of a serendipitous discovery while investigating the scope of the abovementioned aziridine fragmentation chemistry, we found that sulfonyl iodides and bromides react rapidly with [1.1.1]propellane to form a range of sulfonyl BCP halides in as little as 2 min in excellent yields (Scheme 11).^33^ This reaction was suitable for the functionalization of the relatively complex sildenafil and cafenstrole. We were fortunate to be able to collaborate with chemists at Enamine to demonstrate that the reaction could be scaled to near 100 g levels with no detriment to the efficiency.

**Scheme 12:**
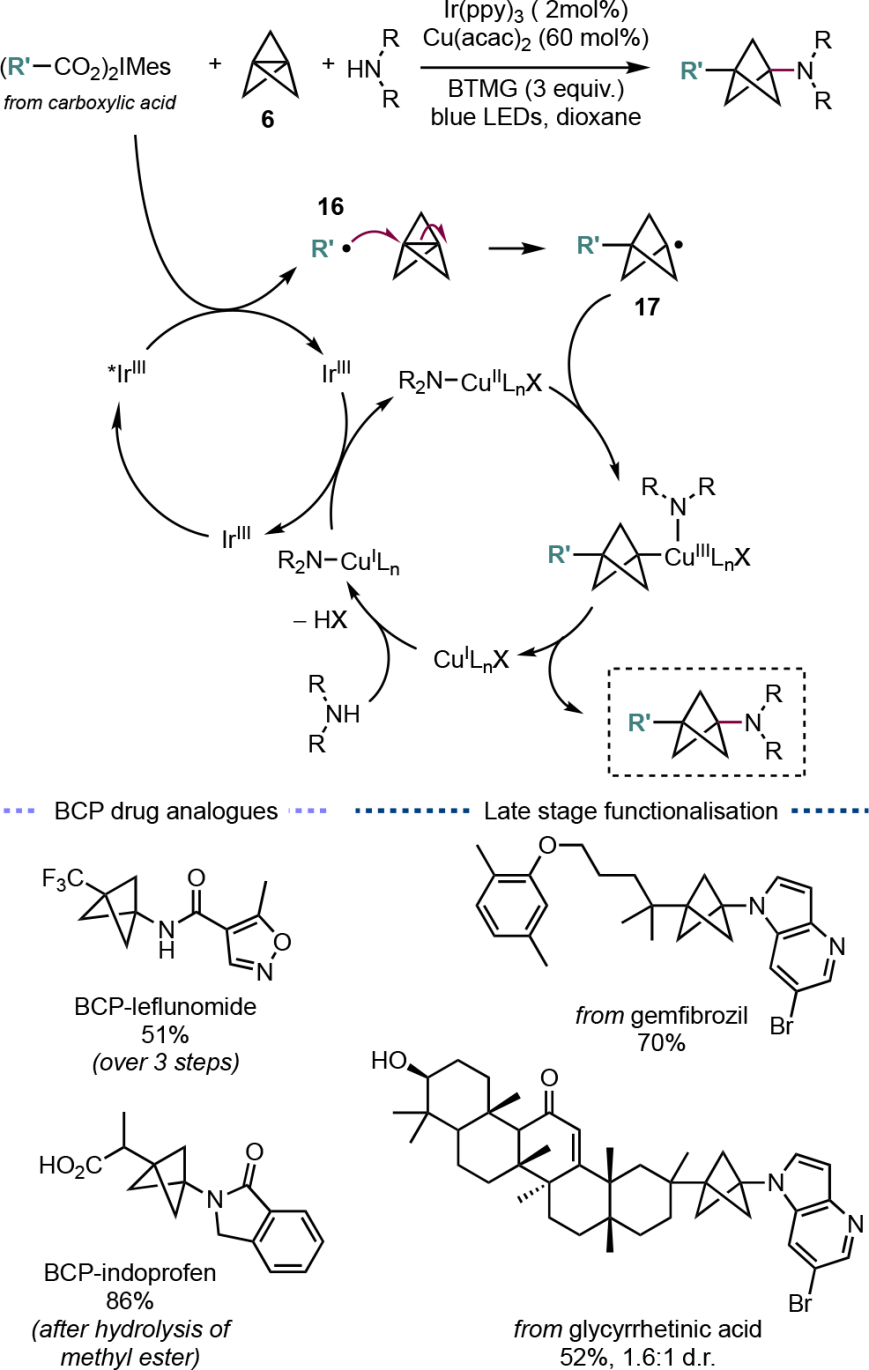
Dual Ir/Cu-Catalyzed Synthesis of *C*,*N*-Functionalized BCPs (MacMillan et al.^34^)

The utility of photoredox-catalyzed methods for the synthesis of BCPs has also been successfully exploited by other groups. For example, the MacMillan group demonstrated a one step *C*,*N*-difunctionalization of **6** with iodonium dicarboxylates and amines using a dual-catalysis system of an iridium photocatalyst and Cu(acac)_2_ (Scheme 12).^34^ The excited-state iridium catalyst can reduce the iodonium carboxylate to form a carbon-centered alkyl radical **16** (upon loss of CO_2_), which can then add to [1.1.1]propellane to give a bridgehead BCP radical **17**. This radical can then be reduced by the amine-ligated copper catalyst, which upon reductive elimination gives the *C*,*N*-difunctionalized BCP product. Through this method, the group was able to rapidly synthesize BCP analogues of the rheumatoid arthritis drug leflunomide (51% yield over 3 steps) and the nonsteroidal anti-inflammatory indoprofen (86% yield over 2 steps). In addition, it proved possible to perform late-stage functionalizations of drug compounds, such as gemfibrozil, and natural products, such as glycyrrhetinic acid.

**Scheme 13:**
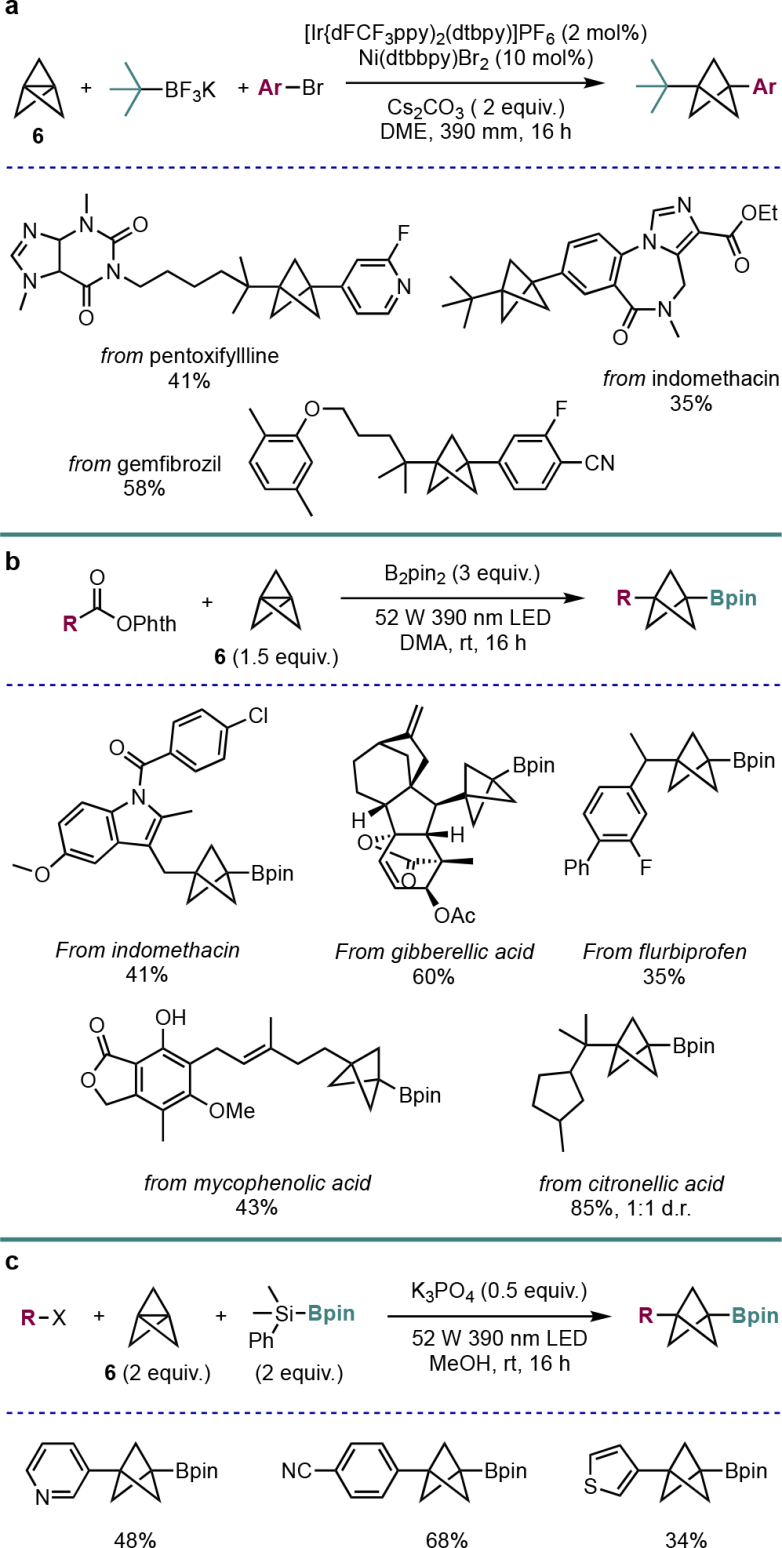
Dual Ir/Ni-Catalyzed Synthesis of *C*,*C*-Functionalized BCPs (Molander et al.^35,36^) via(a) Dual-Catalytic Addition of Alkyl Trifluoroborate Salts to **6**, (b) Photochemical Decarboxylative Borylation of **6** using B_2_pin_2_, and (c) Photochemical Carboborylation of **6** Using Organohalides and PhMe_2_Si–Bpin

The Molander group have also demonstrated the late-stage functionalization of drug compounds with BCPs using a photoredox-catalyzed approach (Scheme 13). Using dual nickel/photoredox catalysis, they were able to achieve dicarbofunctionalization of **6** with tertiary alkyl tetrafluoroborate salts and (hetero)aryl bromides, thereby establishing complex scaffolds in one step (Scheme 13a).^35^

The same group also recently demonstrated a visible-light-mediated difunctionalization of **6** with redox-active esters (Scheme 13b).^36^ Using B_2_pin_2_ as the borylating agent, they were able to prepare a wide variety of BCP-Bpin compounds in one step, including as late-stage functionalizations of many drugs and natural products, in moderate to good yields. This work also included a method for the synthesis of alkyl, aryl, and heteroaryl BCP-Bpins using organohalides and the Suginome borane (PhMe_2_Si–Bpin) under 390 nm LED irradiation (Scheme 13c). Together, these two methods offer a very efficient, transition-metal-free synthesis of BCP boronic esters in a single step, which can then be further functionalized through various methods.

**Scheme 14:**
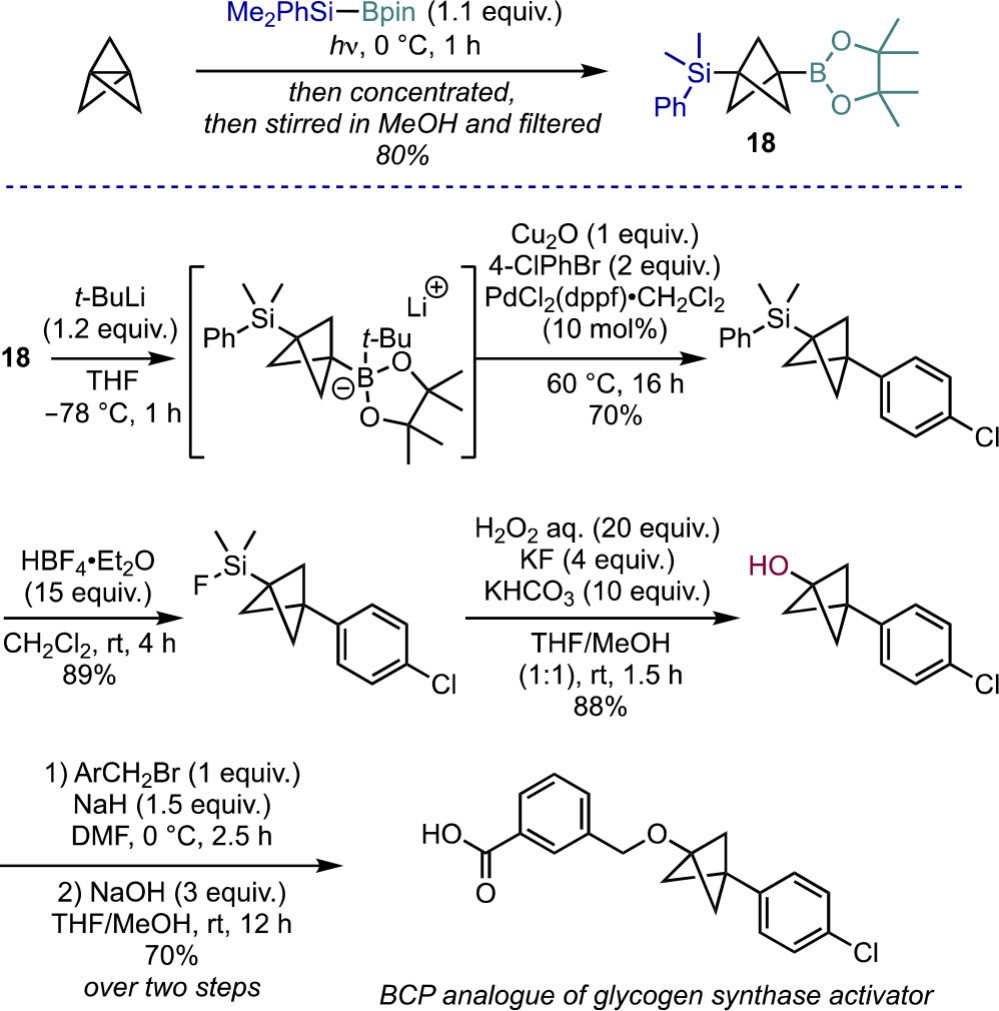
Silylboration of [1.1.1]Propellane and Subsequent Functionalization (Uchiyama et al.^37^)

In contrast to the Molander group’s work, Uchiyama et al. had reported an earlier use of the Suginome borane to achieve the silaboration of [1.1.1]propellane, which formed a desymmetrized and storable BCP (**18**, Scheme 14).^37^ This reaction can be performed on multigram scales, and **18** is reported to be easy to purify. This intermediate bears two distinct functionalizable handles, and its utility was demonstrated in the synthesis of a BCP analogue of a glycogen synthase activator in five steps from intermediate **18** in high yields. The key BCP functionalization steps involved the Suzuki cross-coupling of an ate complex formed by activation with *t*-BuLi, and a Tamao-Fleming oxidation of the phenylsilane to a BCP alcohol.

## Synthesis of Bridge-Substituted BCPs

While a plethora of methods now exist for the synthesis of bridgehead-disubstituted BCPs, mainly because of the facility of the ring-opening of [1.1.1]propellane, as described above, access to bridge-substituted variants is significantly more limited. This is in spite of the value of introducing a new substituent vector on the BCP cage, which offers clear opportunities in drug design. Only recently has a “general” approach been described that begins to address this challenge.

**Scheme 15:**
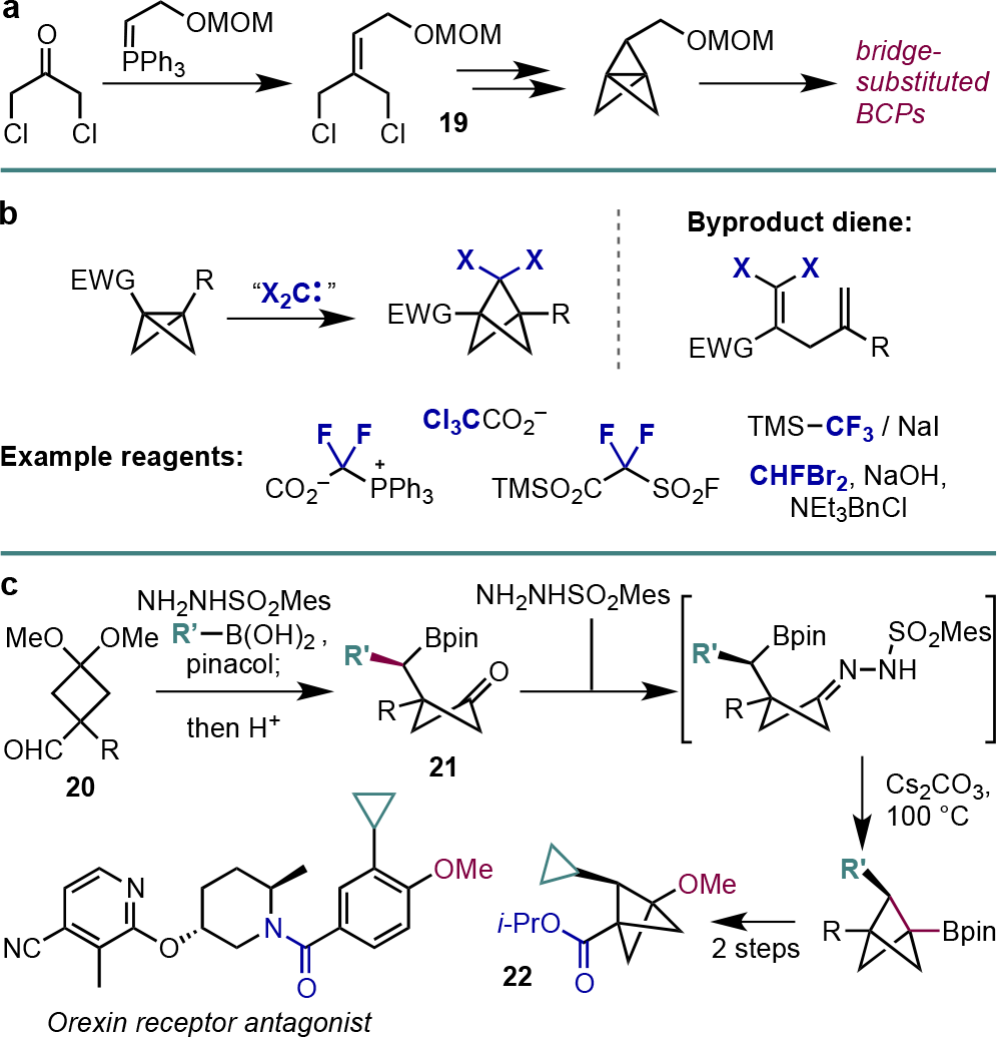
Synthesis of Bridge-Substituted BCPs, including (a) a Classical Route to Bridge-Substituted [1.1.1]Propellanes, (b) Dihalocarbene Insertion in Bicyclo[1.1.0]butanes, and (c) a Barluenga-Type 1,2-Metallation Approach to Bridge-Substituted BCPs (Qin et al.^40^)

Historically, two main methods have been used to introduce bridge substituents. The first involves the synthesis of bridge-functionalized propellanes by variation of the standard path to propellane synthesis. Specifically, the olefination of 1,3-dichloroacetone with various Wittig reagents leads to trisubstituted alkenes, such as **19**,^38^ which afford upon dibromoolefination and cyclization (not shown) the corresponding bridge-substituted propellane (Scheme 15a). The limitations of this chemistry include the challenge of isolation of the propellane, where the higher boiling point of the substituted product has consequences for the usual distillation protocol and ultimately limits the prospects of this strategy. In addition, the substituent selected for bridge substitution must be introduced early in the synthetic sequence, which is less desirable for applications in drug discovery where late-stage diversification is desirable.

The second method to introduce bridge substitution involves the addition of dihalocarbenes to bicyclo[1.1.0]butanes (BCBs). This approach also has its origins in classical routes to nonfunctionalized BCPs, as the addition of dichlorocarbene can be followed by radical-based dehalogenation to afford a methylene bridge (Scheme 15b).^14^

The potential of this chemistry, which avoids propellane-based BCP syntheses, was recognized by GlaxoSmithKline in the synthesis of a BCP analogue of the lipoprotein-associated phospholipase A2 inhibitor darapladib (see below).^15^ Nonetheless, this chemistry suffers from the need for stoichiometric hydrogen atom sources to effect dehalogenation. More recently, attention has turned to the synthesis of difluorinated BCPs via equivalent difluorocarbene insertions, for which a range of carbene precursors have been explored.^39^ Although appealing, this insertion can also suffer from the formation of significant quantities of 1,4-diene byproducts, which arise from a fragmentation pathway during the stepwise carbene addition process. Monofluorination was recently achieved by Mykhailiuk and co-workers using bromofluorocarbene followed by debromination.^6^ Given the prevalence of fluorinated motifs in drug candidates, this chemistry certainly makes a useful addition to the field.

As discussed in this section, the introduction of diverse carbon-based substituents on the BCP bridge is very challenging by propellane-based approaches. An elegant solution to this problem was recently reported by the Qin group, who developed a 1,2-metalate rearrangement strategy to diversify the bridge position.^40^ The concept is outlined in Scheme 15c: a cyclobutane acetal aldehyde **20** is converted to a hydrazone, which undergoes a Barluenga reaction upon treatment with an alkylboronic acid, where addition of a diazoalkane formed *in situ* to the boronic acid triggers migration of the boronic acid substituent. The cyclobutanone **21**, revealed by acid-catalyzed hydrolysis, then in turn undergoes an intramolecular Barluenga reaction upon formation of a second hydrazone, which leads to the formation of the bridge-substituted BCP. This highly modular approach to BCP synthesis was demonstrated in a wide variety of settings, including the formation of trisubstituted BCP **22**, which is a potential building block for the synthesis of orexin receptor antagonist analogues.

## Reactions of Bicyclo[1.1.1]pentanes

Many of the reactions described in the previous sections result in the formation of BCPs bearing “functionalizable” handles, such as halides or boronic esters. Unsurprisingly, many useful manipulations of these groups have been developed in the context of the synthesis or functionalization of BCP analogues of drugs or natural products.

**Scheme 16:**
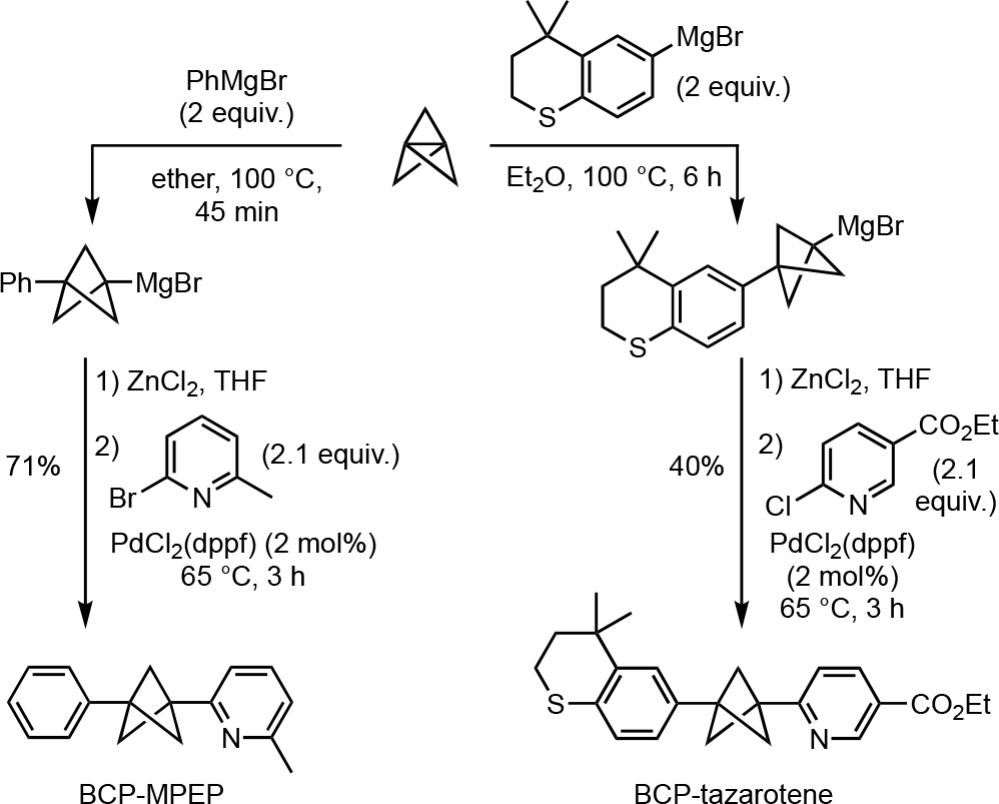
Negishi Cross-Coupling of Bridgehead BCP Organozincs (Knochel et al.^16b^)

The functionalization of BCP Grignards was mentioned earlier in this perspective; however, it is worth revisiting the cross-coupling of these intermediates because of their potential to enable the rapid synthesis of drug analogues (Scheme 16). An example is the Negishi cross-coupling of BCP Grignard reagents developed by Knochel and co-workers, in which transmetalation to an organozinc species is followed by cross-coupling with a range of aryl and heteroaryl halides.^16b^ These transformations are operationally simple, proceed in good yields, and have been applied to the rapid synthesis of bis-arylated BCP analogues of the retinoid tazarotene and the mGluR5 antagonist MPEP.

**Scheme 17:**
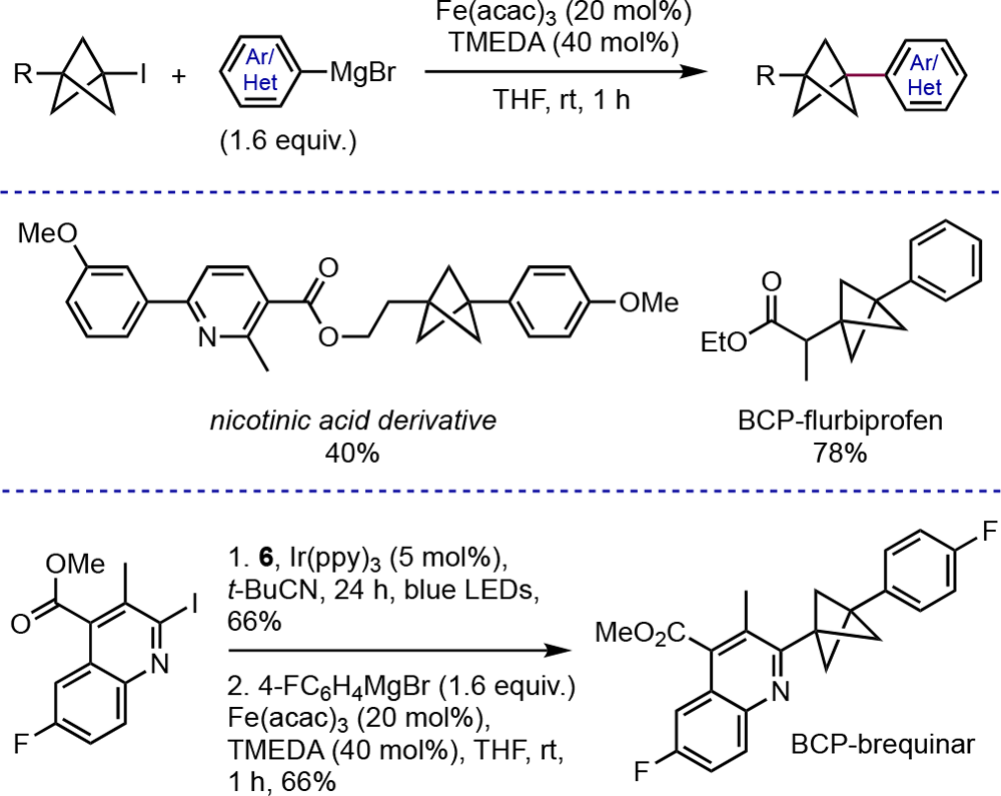
Kumada Cross-Coupling of Bridgehead BCP Iodides (Anderson et al.^41^)

Our group has contributed to the functionalization of BCPs through transformations of bridgehead iodine atoms installed through triethylborane-initiated and photoredox-catalyzed methodologies. In addition to the photoredox-catalyzed Giese reactions of carbon-, nitrogen-, and sulfur-substituted BCP iodides discussed above,^30^ we also developed an iron-catalyzed Kumada cross-coupling of BCP iodides with aryl and heteroaryl Grignard reagents (Scheme 17).^41^ This reaction uses a simple Fe(acac)_3_/TMEDA catalyst system and requires at most one hour at room temperature to reach completion. Using these conditions, we were able to prepare relatively complex BCPs, such as a nicotinic acid derivative, a BCP analogue of the nonsteroidal anti-inflammatory agent flurbiprofen (in just two steps), and a BCP-brequinar analogue (in six steps). This reaction demonstrated the first general direct cross-coupling of BCPs in which the BCP serves as the electrophile component, as well as the first examples of Kumada cross-coupling of tertiary iodides in general.

**Scheme 18:**
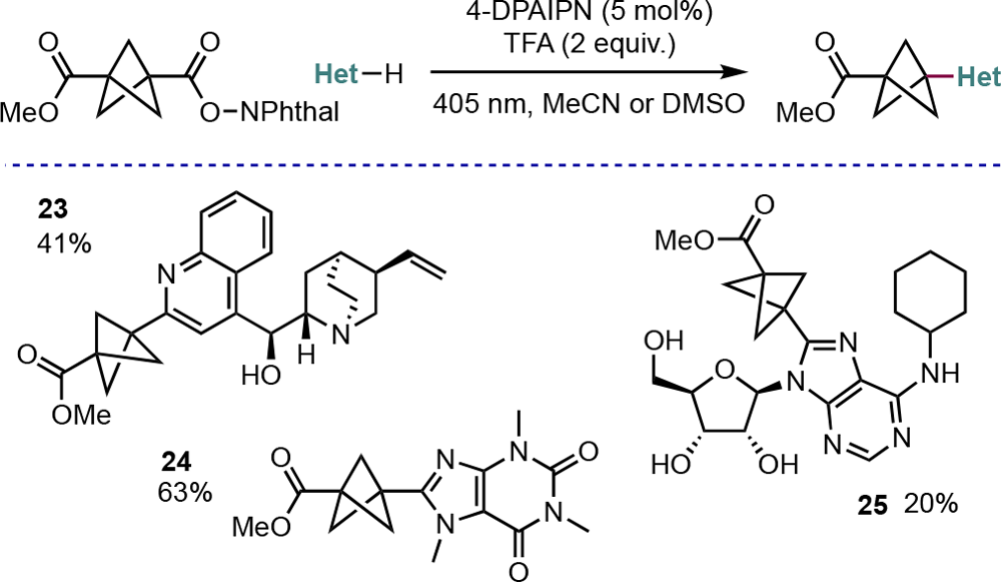
Decarboxylative Minisci Coupling of Redox-Active BCP Esters (Mousseau et al.^42^)

The potential to reform bridgehead radicals from suitable BCP precursors has also been exploited by Mousseau and co-workers, who developed a mild organocatalyzed photo-Minisci reaction between BCPs substituted with redox active esters and heteroarene acceptors (Scheme 18).^42^ An automated flow-based, nanomole-scale high-throughput experimentation/optimization method was deployed to identify optimal reaction conditions, which reduced the amount of BCP substrate needed for reaction screening. This led to the identification of the organic photocatalyst 4-DPAIPN as optimal, which avoids the need for the less sustainable transition metal catalysts often used. Many heteroaryls were suitable for this reaction, including cinchonidine **23**, caffeine **24**, and the unprotected ribonucleic acid **25**, thereby demonstrating the compatibility of this reaction with late-stage functionalization.

**Scheme 19:**
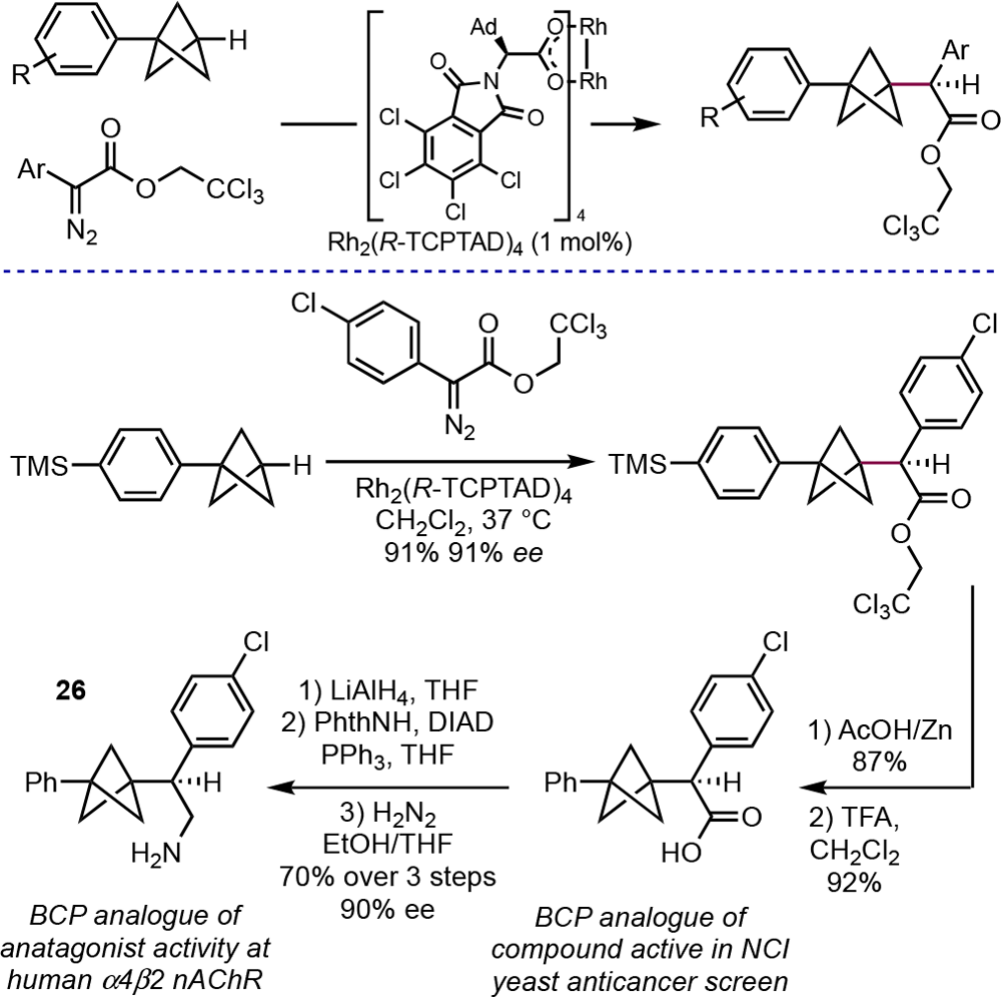
Rhodium-Catalyzed Bridgehead C–H Diazocarbonyl Insertion (Davies et al.^43^)

The BCP derivatives discussed above are equipped with groups that enable further functionalization, such as iodides and redox active esters. Equally appealing are chemistries to functionalize BCPs that do not require such substituents and enable direct reaction with C–H bonds. Although many reactions have been developed to form terminal, monosubstituted BCPs, the functionalization of the bridgehead C–H bond remains challenging. Davies and co-workers have addressed this problem through the development of a rhodium-catalyzed enantioselective bridgehead C–H functionalization using donor/acceptor carbenes (Scheme 19).^43^ This method proceeded in very good yields and enantioselectivity, albeit requiring an arene substituent at the other bridgehead position, and could be applied to the synthesis of a BCP analogue of a human α4β2 nAChR antagonist **26**.

**Scheme 20:**
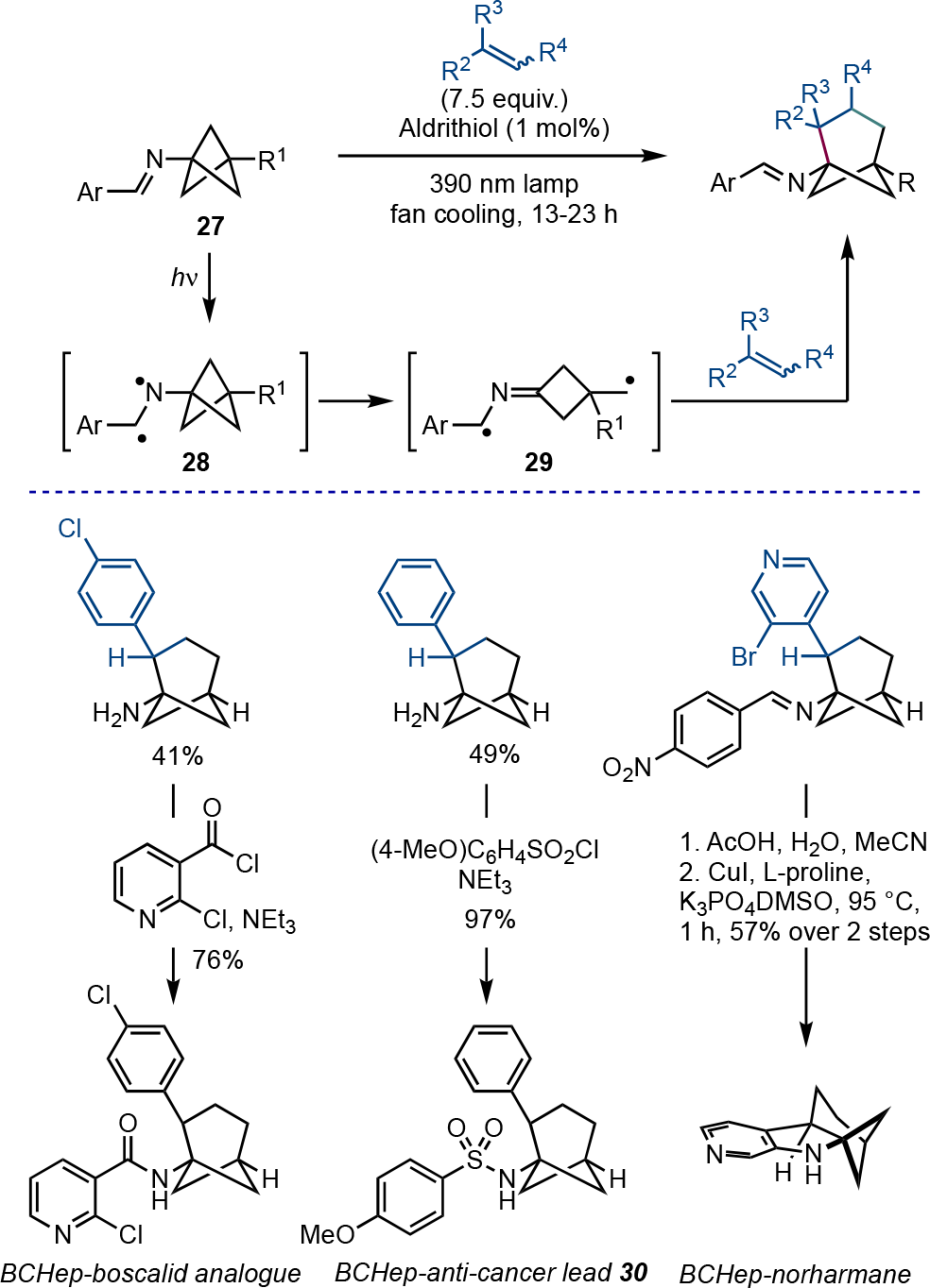
Photochemical Alkene Insertion into BCP Imines (Stephenson et al.^44^)

An unusual mode of BCP reactivity is ring expansion of the BCP bridge to larger bicycloalkanes. This concept has been explored by the Stephenson group, who described a photochemical formal [4 + 2] reaction to form bicyclo[3.1.1]heptanes (BCHeps) from BCP imines **27** (Scheme 20).^44^ In the presence of an excess of an alkene, excitation of the imine generates a diradical **28**, which undergoes fragmentation of the BCP to the diradical **29**. The primary radical thus generated can add to the alkene, and subsequently, cyclization delivers the BCHep product. The bridge substitution in the products makes this a particularly valuable reaction, as this can be difficult to achieve through other means. The reaction was applied to the synthesis of BCHep analogues of a number of natural products and drugs, where the BCHep serves as a surrogate for an *ortho*-substituted benzene ring; examples included boscalid, anticancer lead **30**, and norharmane.

## Applications of Bicyclo[1.1.1]pentanes

By far the most popular use of bicyclo[1.1.1]pentanes lies in medicinal chemistry, where the BCP can serve as a bioisostere for 1,4-disubstituted arenes, alkynes, and *t*-butyl groups. The explosion of interest in the synthesis and reaction of BCPs over the past decade has led to an extensive synthetic toolkit, which has rendered the synthesis of such analogues significantly more facile; this has allowed several groups to synthesize BCP drug analogues and to evaluate their pharmacokinetic properties. A selection of examples are presented here.

**Figure 2:**
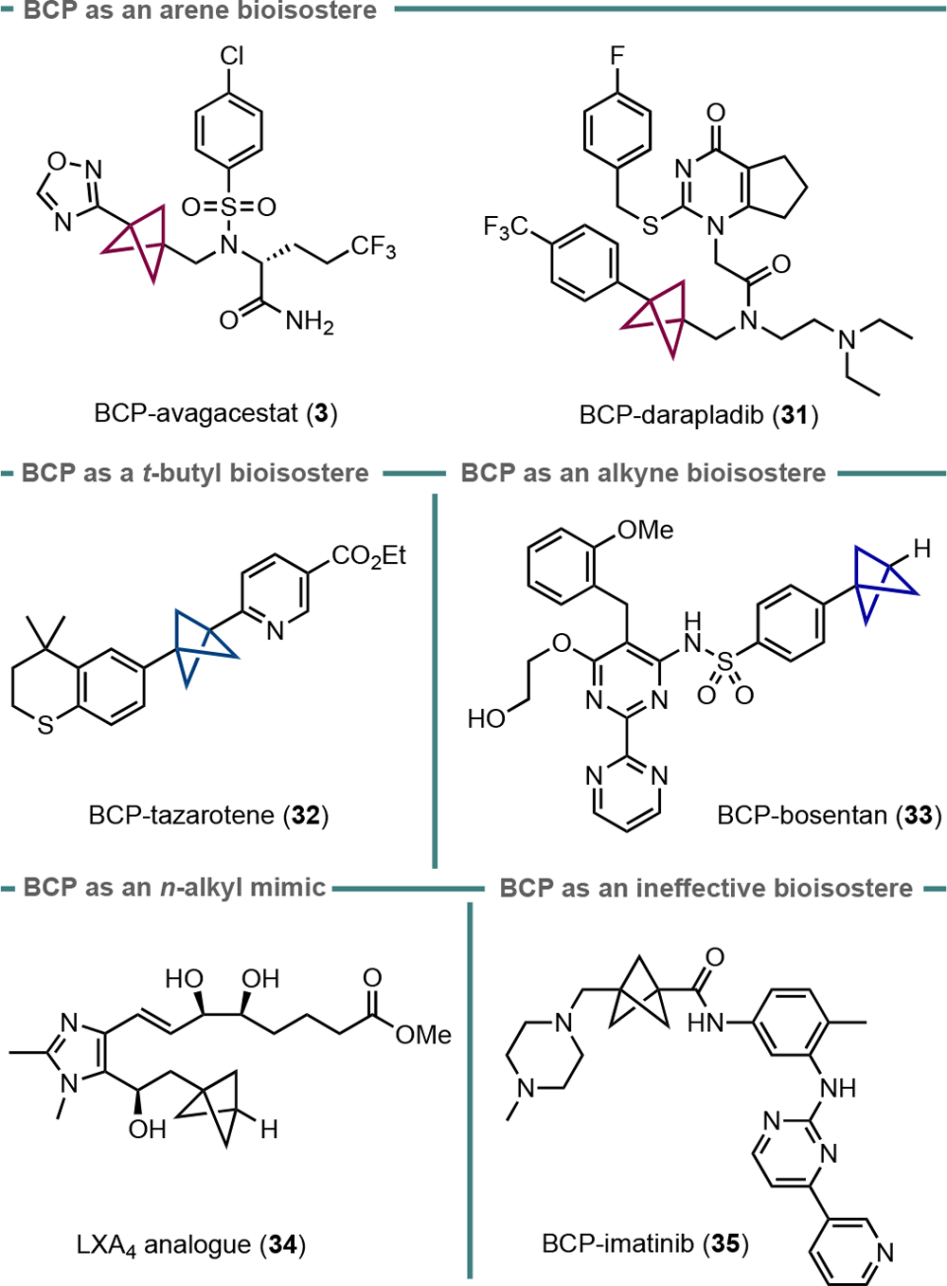
Examples of BCP substitution in bioactive molecules.

Aside from early work by Pellicciari and co-workers, the groundbreaking example of this concept is arguably that of a BCP as a bioisostere for a fluorophenyl moiety in the γ-secretase inhibitor avagacestat described by Stepan et al. (analogue **3**, Figure 2).^45^ Analogue **3** exhibited similar inhibition levels to the parent compound while improving aqueous solubility and passive permeability, therefore leading to significantly higher levels of oral absorption. This effect was attributed to the disruption of both the planarity of avagacestat and the π-stacking of its aromatic rings between molecules. Similar effects were observed by Measom et al. in the BCP analogue **31** of darapladib,^15^ which also exhibited improved permeability and solubility compared with the parent compound.

Both darapladib and avagacestat used a BCP as an arene bioisostere. Knochel and co-workers also demonstrated its successful application in replacing an internal alkyne in the skin-treatment agent tazarotene (**32**), which showed a slightly increased chromatographic hydrophobicity index on immobilized artificial membranes.^16b^ Replacement of a *t*-butyl group with a BCP was assessed by Carreira and co-workers, who surveyed a range of possible isosteres for the *t*-butyl group of the dual endothelin receptor antagonist bosentan,^46^ used for treatment of pulmonary arterial hypertension. In this case, the BCP analogue **33** showed very similar solubility and permeability to the parent drug, but also displayed a large decrease in the IC_50_ value for human endothelin receptor subtype B, thereby indicating a significant increase in activity compared with the parent bosentan. More recently, Guiry and co-workers reported the synthesis and evaluation of BCP-containing aromatic lipoxin A_4_ analogues as metabolically resistant bioisosteres for alkyl chains in fatty-acid-derived molecules.^47^ They discovered that of four BCP-containing mimetics synthesized, one (**34**) did indeed possess high anti-inflammatory activity.

While there are many examples of BCP analogues of drugs showing improved pharmacokinetic properties and maintaining potency, this is not always the case. Nicolaou, Stepan, and co-workers investigated a BCP analogue of the anticancer drug imatinib (**35**). While solubility was significantly improved, potency decreased by around 80-fold;^48^ molecular docking calculations suggested this loss of potency could be due to the shorter length of the BCP linker compared with the arene in the parent imatinib, which led to disruption of key hydrogen bonding interactions between the drug and the kinase. Overall, these studies underline the relatively consistent ability of BCPs to improve the solubility of drug compounds; however, other properties, such as metabolic stability and potency, are less easy to predict, and it would be useful to expand understanding in this area for the synthesis and evaluation of further BCP analogues in the future. Possible directions for study include the use of BCP fragments as motifs in fragment-based drug discovery, as well as the use of BCPs as distinct rigid scaffolds in their own right.

**Scheme 21:**
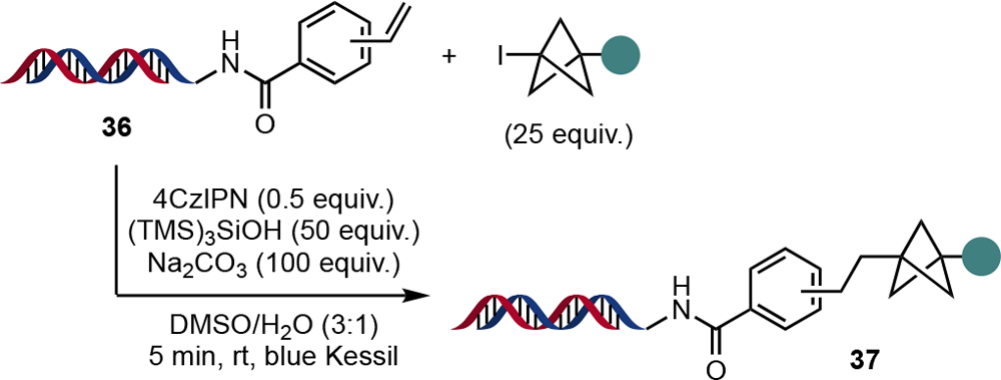
Synthesis of DNA-Encoded Libraries by Giese Reaction of BCP Iodides (Molander et al.^49^)

Beyond the synthesis of pharmaceutical analogues, Molander, Crane, and co-workers have demonstrated the use of BCPs to access DNA-encoded libraries (DELs) with a greater fraction of C(sp^3^) content (Scheme 21).^49^ BCP iodides were subjected to photocatalyzed Giese reactions with DNA headpiece **36** as a radical acceptor to form products **37**. This chemistry proved less damaging to the DNA tags than other DEL chemistry, with DNA damage assessment showing good retention of PCR amplification ability and only 6% of mutated sequences observed for a full-length DNA tag.

**Figure 3:**
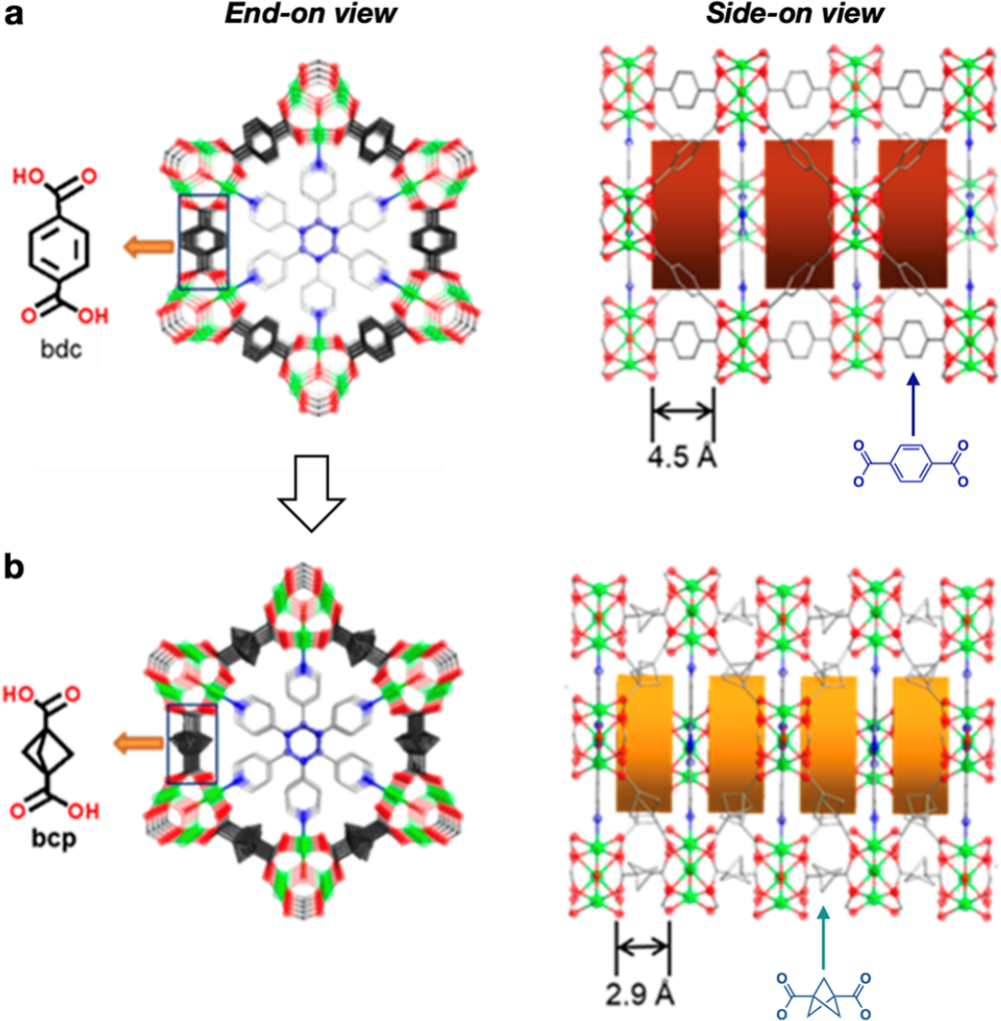
Use of BCP-dicarboxylic acid as a ligand in covalent organic framework (COF) design. (a) Depiction of the benzene dicarboxylate-linked COF and (b) depiction of the BCP dicarboxylate-linked COF. Reproduced with permission from ref (50). Copyright 2022 American Chemical Society.

Finally, a highly innovative use of BCPs has recently been demonstrated in pore-space partitioning by Feng and Bu et al. (Figure 3).^50^

Using benzene-1,4-dicarbarboxylic acid (terephthalate) as a design basis, hexagonal porous material could be constructed by trimerization with various metal ions (end-on view, Figure 3a). The channels in these pores can be occupied by polypyridine guests, with each hexagonal array separated from the next by the dicarboxylate ligand (side-on view). Substitution of terephthalate with the shorter BCP-dicarboxylic acid ligand resulted in a structure containing ultramicropores that exhibited enhanced selectivity between C_2_H_2_/CO_2_ and C_3_H_6_/C_3_H_8_ (Figure 3b). This selectivity was especially notable for the latter pair in which a large gap between their adsorption isotherms is observed for the BCP analogue and not the original terephthalate compound: ideal adsorbed solution theory selectivity calculations showed a 177% increase in this selectivity. Given the diversity of BCP and related scaffolds now available, this remarkable application of BCPs suggests much opportunity beyond medicinal chemistry.

## Future Directions

The technologies outlined in this perspective now arguably offer the ability to access almost any bridgehead-substituted BCP. Nonetheless, challenges remain for the BCP field: foremost among these is the need to solve the “propellane problem”—the inevitable reliance of most methodologies on the direct use of the volatile and reactive [1.1.1]propellane, which presents difficulties in both synthesis and storage. Even the availability of presynthesized BCP building blocks merely masks this issue in an earlier synthetic stage and currently may well prevent implementation of the BCP motif on the process scale. A “green” BCP synthesis is, therefore, much in demand!

**Scheme 22:**
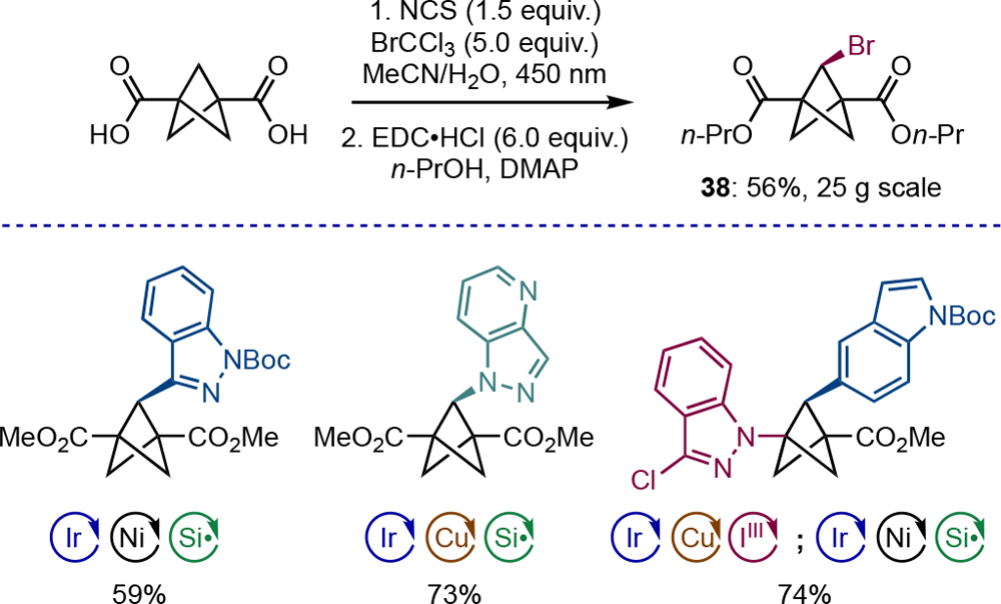
Divergent Synthesis of Bridge-Functionalized BCPs (MacMillan et al.^51^)

Bridge functionalization that avoids lengthy synthetic sequences is also a significant limitation that remains to be addressed. A recent report from MacMillan and co-workers offers a first breakthrough in this field (Scheme 22):^51^ bromination of BCP dicarboxylic acid was achieved on the multigram-scale using BrCCl_3_ as a bromine atom source with bridgehead H atom abstraction mediated by photochemically generated chlorine radicals. Using the methyl ester analogue of brominated product **38**, an impressive range of C–C and C–N bond formations were carried out using dual-catalysis conditions developed previously by the group. Impressively, both the bridge and bridgehead sites could be functionalized by taking advantage of decarboxylative coupling of iodonium dicarboxylates.^52^

**Scheme 23:**
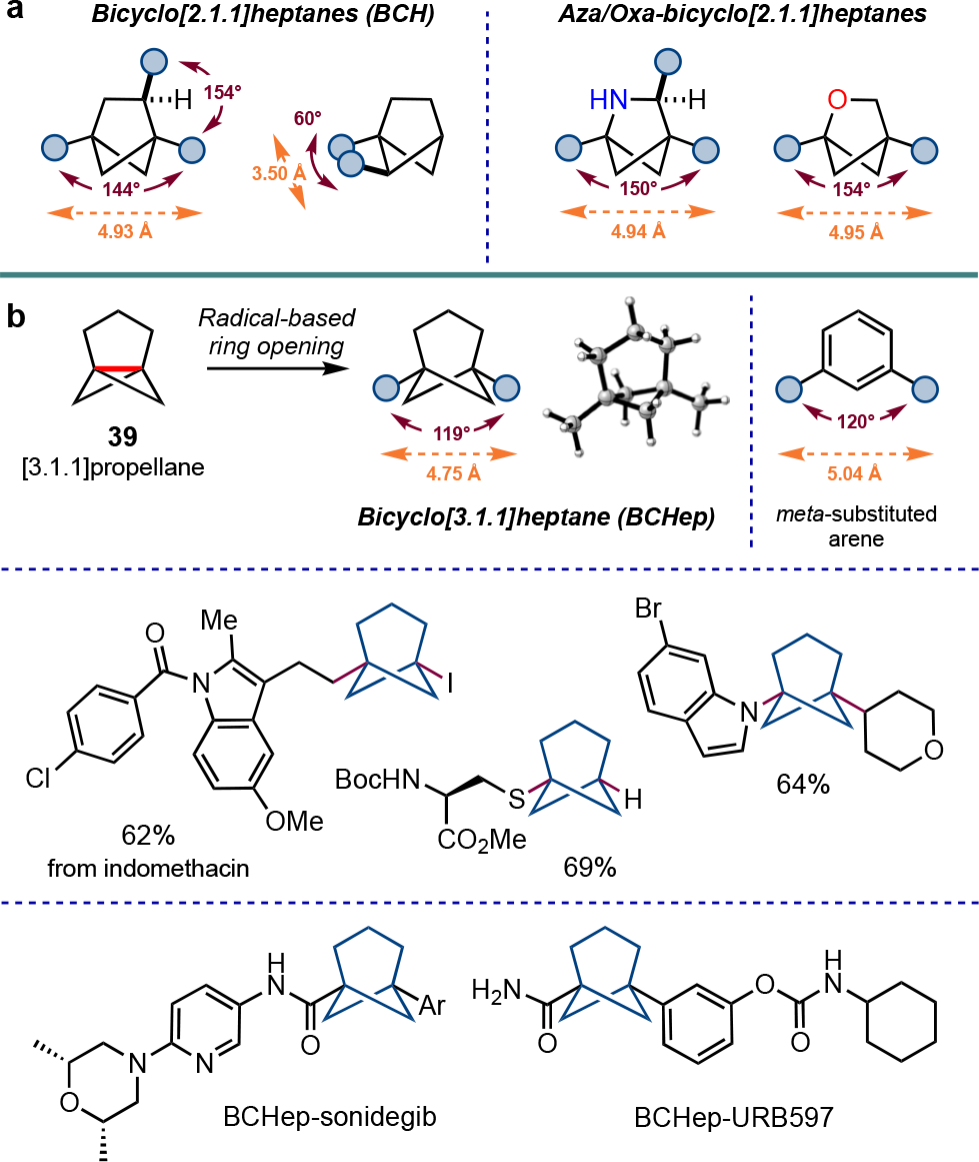
(a) Related Bicyclic Scaffolds of Interest in Medicinal Chemistry Research and (b) Radical-Based Synthesis of Bicyclo[3.1.1]heptanes (BCHeps) (Anderson et al.^55^)

Alongside this challenge, the field of rigid cage (bio)isosteres is gravitating toward the use of such structures not only as mimics of benzene rings but as rigid scaffolds in their own right that offer the potential to position substituents along well-defined vectors. This has led to a repopularization of the bicyclo[2.1.1]hexane (BCH) motif^53^ and heteroatom variants,^54^ where some elegant chemistries have recently been described (Scheme 23a). Our group has recently disclosed a scalable access to [3.1.1]propellane **39**, which opens up convenient radical-based access to bicyclo[3.1.1]heptanes (BCHeps)—scaffolds that faithfully reproduce the geometric properties of *meta*-substituted benzene rings and also offer some of the same physicochemical and pharmacokinetic benefits as their BCP cousins (Scheme 23b).^55^ Even this structure presents opportunities for further research, such as overcoming the challenge of the addition of anionic reagents to **39**, which has proven effective for [1.1.1]propellane. For both BCPs and BCHeps, the ready availability of heteroatom-containing analogues is also an attractive but unsolved challenge, while further improving the efficiency of bridge functionalization will offer many opportunities for applications in biological contexts.

In all, the chemistries developed over the past decade have truly conquered the synthesis of bicyclo[1.1.1]pentanes, thereby making available a veritable plethora of structures for applications in medicinal chemistry and beyond. Challenges and opportunities remain, which will no doubt continue to inspire the synthetic community over the coming years!
